# DRB2 Is Required for MicroRNA Biogenesis in *Arabidopsis thaliana*

**DOI:** 10.1371/journal.pone.0035933

**Published:** 2012-04-24

**Authors:** Andrew L. Eamens, Ki Wook Kim, Shaun J. Curtin, Peter M. Waterhouse

**Affiliations:** 1 School of Molecular Bioscience, University of Sydney, Sydney, New South Wales, Australia; 2 Department of Agronomy and Plant Genetics, University of Minnesota, St. Paul, Minnesota, United States of America; 3 School of Biological Sciences, University of Sydney, Sydney, New South Wales, Australia; 4 CSIRO Plant Industry, Canberra, Australian Capital Territory, Australia; Ecole Normale Superieure, France

## Abstract

**Background:**

The *Arabidopsis thaliana* (*Arabidopsis*) DOUBLE-STRANDED RNA BINDING (DRB) protein family consists of five members, DRB1 to DRB5. The biogenesis of two developmentally important small RNA (sRNA) species, the microRNAs (miRNAs) and *trans*-acting small interfering RNAs (tasiRNAs) by DICER-LIKE (DCL) endonucleases requires the assistance of DRB1 and DRB4 respectively. The importance of miRNA-directed target gene expression in plant development is exemplified by the phenotypic consequence of loss of DRB1 activity (*drb1* plants).

**Principal Findings:**

Here we report that the developmental phenotype of the *drb235* triple mutant plant is the result of deregulated miRNA biogenesis in the shoot apical meristem (SAM) region. The expression of *DRB2*, *DRB3* and *DRB5* in wild-type seedlings is restricted to the SAM region. Small RNA sequencing of the corresponding tissue of *drb235* plants revealed altered miRNA accumulation. Approximately half of the miRNAs detected remained at levels equivalent to those of wild-type plants. However, the accumulation of the remaining miRNAs was either elevated or reduced in the triple mutant. Examination of different single and multiple *drb* mutants revealed a clear association between the loss of DRB2 activity and altered accumulation for both the elevated and reduced miRNA classes. Furthermore, we show that the constitutive over-expression of *DRB2* outside of its wild-type expression domain can compensate for the loss of DRB1 activity in *drb1* plants.

**Conclusions/Significance:**

Our results suggest that in the SAM region, DRB2 is both antagonistic and synergistic to the role of DRB1 in miRNA biogenesis, adding an additional layer of gene regulatory complexity in this developmentally important tissue.

## Introduction

In *Arabidopsis thaliana* (*Arabidopsis*), the various classes of endogenous 21–24 nucleotide (nt) small RNA (sRNA) are processed from structurally distinct double-stranded RNA (dsRNA) precursors by four DICER-LIKE (DCL) proteins, an RNase III-like family of endonucleases [1], [2]. Two functionally well-characterized sRNA classes, the microRNAs (miRNA) and *trans*-acting small-interfering RNAs (tasiRNAs), both of which regulate the expression of developmentally important genes, require DCL1 and DCL4 for their biogenesis [3], [4], [5], [6]. The precise and efficient processing of these dsRNA substrates by DCL1 and DCL4 is mediated by HYPONASTIC LEAVES1 (HYL1; referred to as DRB1 from here on) and DRB4 activity respectively, two of the five members of the *Arabidopsis* dsRNA BINDING (DRB) protein family [7], [8], [9], [10], [11]. The functional importance of these two DCL/DRB protein partnerships on plant development is exemplified by the phenotypes displayed by *dcl1*, *dcl4*, *drb1* and *drb4* plants [7], [9], [12], [13].

In miRNA biogenesis, the primary-miRNA (pri-miRNA) transcript, a long non-protein-coding RNA containing a region of self-complementarity allowing for stem-loop formation, is processed by DRB1-assisted DCL1 in specialized nuclear dicing bodies (D-bodies) [14], [15], [16]. The initial cleavage step of the miRNA biogenesis pathway produces a smaller stem-loop intermediate, the precursor-miRNA (pre-miRNA). The DCL1/DRB1 partnership also directs the second cleavage step of the miRNA biogenesis pathway to liberate the miRNA/miRNA* duplex from the pre-miRNA stem-loop sequence [7], [12], [17]. The 3′ 2-nt overhang of each duplex strand is methylated by the sRNA-specific methyltransferase HUA ENHANCER1 (HEN1) and exported to the cytoplasm where the miRNA guide strand is separated from the miRNA* passenger strand [3], [18]. The liberated miRNA sRNA is loaded onto the ARGONAUTE1 (AGO1)-catalyzed RNA-induced silencing complex (RISC) to predominantly direct cleavage of highly complementary mRNAs [13], [19].

In the closely related tasiRNA biogenesis pathway, three miRNAs, miR173, miR390 and miR828, guide AGO1 (miR173 and miR828) or AGO7 (miR390)-catalyzed cleavage of *TAS* transcripts, long non-protein-coding RNAs transcribed from *TAS* loci [20], [21], [22]. This identifies the cleaved *TAS* transcript as a template for dsRNA synthesis via the combined action of SUPPRESSOR OF GENE SILENCING3 (SGS3) and RNA-DEPENDENT RNA POLYMERASE6 [9], [23]. The resulting dsRNA is processed by DCL4 with the assistance of DRB4 into phased 21-nt tasiRNA/tasiRNA* duplexes. Following the methylation of duplex strands, and export to the cytoplasm, the tasiRNA guide strand is loaded by AGO1-catalyzed RISC to direct cleavage-based RNA silencing of cognate mRNAs in *trans* [5], [22], [24].

Unlike the DRB requirements of DCL1 and DCL4, the production of 22 and 24-nt sRNAs from structurally distinct dsRNA substrates by DCL2 and DCL3 was thought to be DRB-independent. Our previous analyses were unable to detect any alteration in the accumulation of DCL2 or DCL3-specific sRNAs, or the expression of their respective target genes in any *drb* mutant plant line assessed [25]. This suggested that DRB2, DRB3 and DRB5 are not involved in sRNA biogenesis or target gene expression regulation. However, it has recently been shown that DRB2 is antagonistic to DRB4 in the production of all siRNA size classes from RNA polymerase IV (PolIV) derived transcripts [26]. This finding, and similarities in the abnormal development of the *drb235* triple mutant and that of plant lines defective for miRNA pathway components, including *dcl1* hypomorphic and *drb1* null alleles [7], [13], [27] led us to investigate the roles of DRB2, DRB3 and DRB5 in miRNA biogenesis. In seedlings of wild-type *Arabidopsis* plants, the expression profiles of *DRB2*, *DRB3* and *DRB5* overlap in the tissues of the shoot apical meristem (SAM) region. In this region of *drb235* plants, sRNA sequencing revealed that accumulation of different miRNA classes was either enhanced, unchanged or reduced. Molecular analyses revealed a clear association between the loss of DRB2 activity and the altered accumulation of both the elevated and reduced miRNA classes. This shows that DRB2 is both antagonistic and synergistic to DRB1 in miRNA biogenesis in this developmentally important tissue. We further demonstrate, through complementation of the *drb1* mutant by constitutively over-expressing a *DRB2* transgene that the involvement of DRB2 in miRNA biogenesis is restricted by its tissue-specific expression in wild-type *Arabidopsis* plants.

## Results

### DRB family member expression and mutant phenotypes

**Figure 1:**
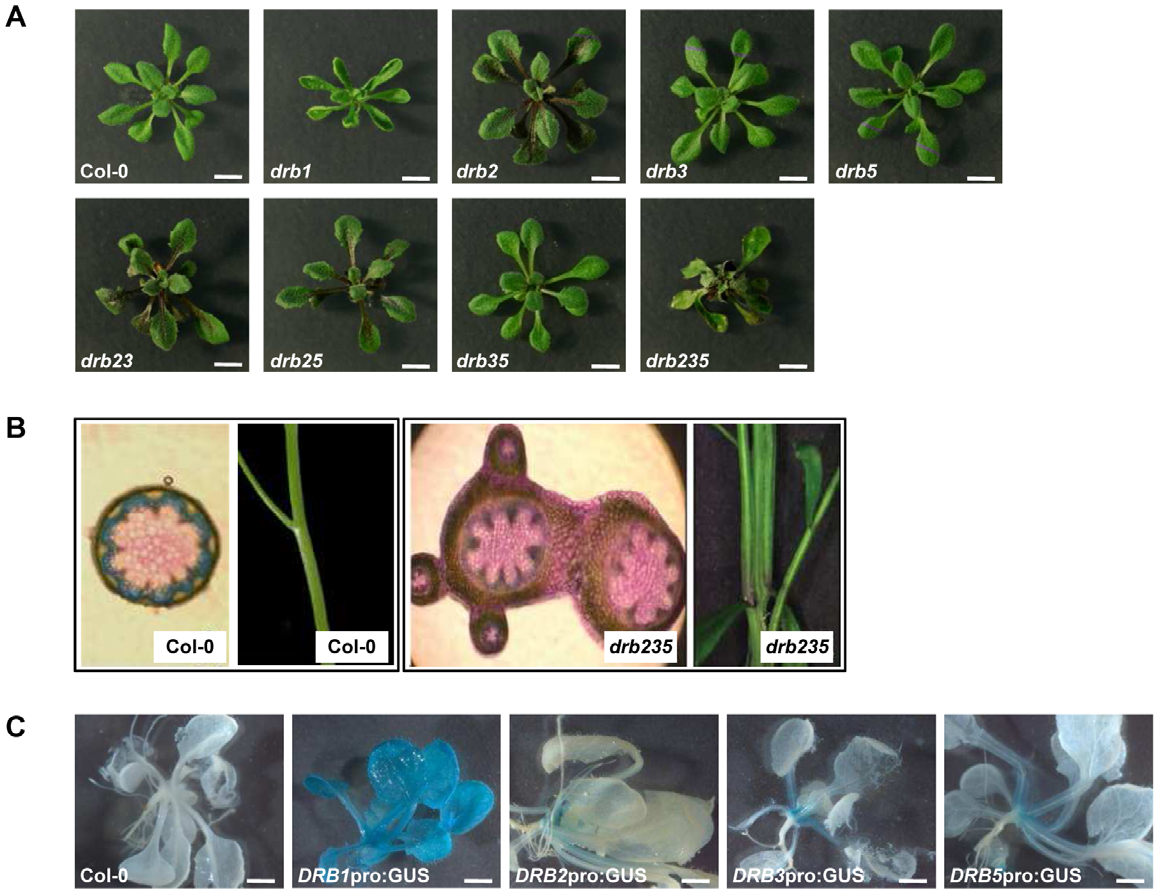
*DRB* expression and mutant phenotypes. (A) Vegetative phenotypes expressed by 4 week old *drb* T-DNA knockout mutant lines. Scale bars = 5 mm. (B) Fusion of the inflorescence stem of *drb235* plants. (C) GUS expression localized to the SAM region of *DRB2*pro:GUS, *DRB3*pro:GUS and *DRB5*pro:GUS plants. Scale bars = 2 mm.

In *Arabidopsis*, miRNAs typically require the DCL1/DRB1 protein complex for their biogenesis [4], [22]. In accordance with reduced mature miRNA accumulation, *drb1* plants express a pleiotropic phenotype, characterized by upwardly curled (hyponasty) rosette leaves, and reduced organ size, growth and fertility [7], [12], [25], [28]. The loss of DRB2 also results in changes to rosette leaf morphology (Figure 1A). Compared to wild-type plants, *drb2* rosette leaves are ovoid, flatter and darker in color due to increased anthocyanin production. As *drb2* plants mature, the margins of their rosette leaves become highly serrated. Furthermore, the *drb2* phenotype is epistatic to those expressed by *drb3* and *drb5* plants. Plants defective for the activity of these two DRB family members are essentially wild-type in appearance, as is the double mutant *drb35* (Figure 1A).

Our previous analyses demonstrated that DRB2, DRB3 and DRB5 are highly similar at both the genomic and amino acid level [25] suggesting that these three family members may be functionally redundant. As described for the *drb1* plant line with reduced miRNA accumulation, the *drb235* triple mutant also expresses a pleiotropic phenotype. Although the margins of *drb235* rosette leaves remain serrated, they do not display the dark-green coloration and ovular shape of *drb2* rosette leaves. Instead, they are pale green in color with dark green venation of the vasculature, have a lanceolate shape and are downturned at their tips (epinasty). Furthermore, the petioles of *drb235* rosette leaves are markedly shorter than those of wild-type plants, and this combined with their downward curvature leads to the formation of a compact rosette (Figure 1A). In contrast to all other single, double or triple *drb* mutant plants, which essentially display the architecture of a wild-type inflorescence stem, the stems of *drb235* plants are fused (Figure 1B).

Consistent with its role as a cofactor for DCL1 in miRNA biogenesis, *DRB1*pro:GUS plants showed constitutive and ubiquitous reporter gene expression. GUS expression was restricted to the SAM region of plants transformed with the *DRB3*pro:GUS and *DRB5*pro:GUS vectors (Figure 1C). Analysis of plants transformed with our original *DRB2*pro:GUS vector, containing a 1.7 kb putative promoter fragment, suggested that; i) *DRB2* expression was restricted to developing anthers, pollen and germinating seed, and; ii) *DRB2* expression did not overlap with *DRB3* and *DRB5* [25]. However, extending the putative promoter region to include 4 kb upstream of the transcription start site of *DRB2* showed that in vegetative tissue *DRB2* expression is concentrated in the SAM region, an expression domain that overlaps with *DRB3* and *DRB5* (Figure 1C).

### miRNA accumulation in the SAM region of *drb235* plants

The developmental phenotypes expressed by *drb1* and *drb235* plants, in addition to the overlapping expression of *DRB2*, *DRB3* and *DRB5* led us to investigate miRNA accumulation in whole plant samples for comparison to the specific tissues where these three DRB family members are expressed. Alterations to leaf shape, curvature and margin serration have been associated with expression changes to members of several well-characterized miRNA gene (*MIR* gene) families or their target genes [29], [30], [31], [32]. Northern blotting and RT-PCR were used to determine if one, or all three DRB proteins are involved in miRNA biogenesis or action in whole plants. However, these analyses performed on the aerial tissue of 4 week old *drb* mutant plants failed to identify any significant changes to either mature miRNA accumulation (Figure S1A), or expression of their cognate targets (Figure S1B).

We next used sRNA sequencing to quantify mature miRNA levels in the SAM region of *drb235* plants. Selection of this tissue was determined by reporter gene expression analysis of plants transformed with the *DRB2*pro:GUS, *DRB3*pro:GUS and *DRB5*pro:GUS vectors (Figure 1C). Following normalization (normalized to the total number of 20–24 nt sRNA reads mapping to the *Arabidopsis* genome for each sample), sequencing identified 440895 and 307153 sRNA reads in the Col-0 and *drb235* samples respectively that perfectly matched 140 of the 189 mature miRNA sequences entered into the miRBase database (http://www.mirbase.org/) at the time of analysis (Table S1). The 140 mature miRNA sequences detected in both the Col-0 and *drb235* samples by sRNA sequencing represented 57 *MIR* gene families (Table S2). When individual family member reads were combined to give an overall *MIR* gene family score, miRNA accumulation for 8 (14.0%), 26 (45.6%) and 23 (40.4%) *MIR* gene families was elevated, at approximate wild-type levels and reduced respectively in the SAM region of *drb235* plants (Table S3). Table 1 shows the five *MIR* gene families in *drb235* plants with the most significantly elevated or reduced accumulation in the specific tissues assessed by sRNA sequencing. Five miRNAs with unchanged accumulation in *drb235* plants are also listed in Table 1 and were included as wild-type controls. Northern blotting was used to confirm the accumulation profile of each of the 15 miRNAs listed in Table 1 (Figure S2). Three miRNAs, including miR164, miR168 and miR169, all of which form highly conserved, well-characterized *MIR* gene families were selected as representatives for further analysis of the elevated, unchanged and reduced miRNA accumulation classes in *drb235* plants.

**Table 1:** MiRNA accumulation in the SAM region of *drb235* plants.

*MIR* gene	Number of reads		Fold change
family	Col-0	*drb235*	(+/−)
***Elevated miRNA accumulation class***			
*MIR863*	19	469	+24.7
*MIR850*	23	469	+20.4
*MIR837*	24	245	+10.2
*MIR841*	11	96	+8.7
*MIR164* 1	435	3315	+7.6
***Unchanged miRNA accumulation class***			
*MIR162*	4977	5071	1.0
*MIR165*	11331	11718	1.0
*MIR168* 2	408	394	1.0
*MIR319*	6408	6148	1.0
*MIR390*	1196	1244	1.0
***Reduced miRNA accumulation class***			
*MIR822*	929	62	−15.0
*MIR839*	58	6	−9.7
*MIR173*	26	3	−8.7
*MIR169* 3	3796	637	−6.0
*MIR170*	60	11	−5.5

Fold changes of the five *MIR* gene families with the most highly elevated or reduced accumulation in the SAM region of *drb235* plants. Five *MIR* gene families with wild-type accumulation are also listed. *MIR* gene family accumulation was classed as being elevated or reduced in *drb235* plants if the fold change was greater than ±2.0.

1 elevated miRNA class representative.

2 unchanged miRNA class representative.

3 reduced miRNA class representative.

### In the SAM region miRNA accumulation requires DRB2 activity

**Figure 2:**
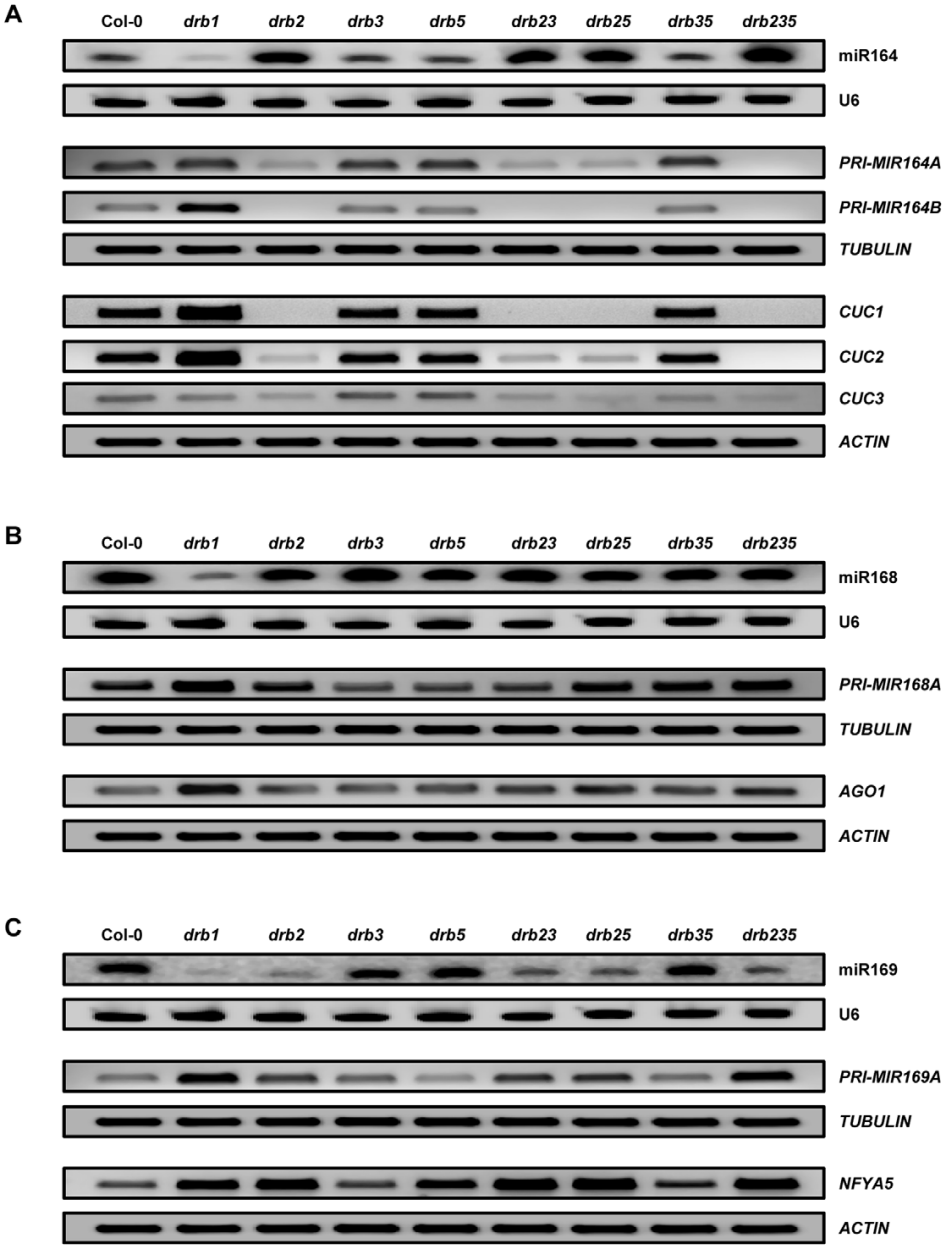
MiRNA accumulation and target gene expression in the SAM region of *drb* mutants. (A) Accumulation of the elevated *drb235* miRNA class representative, miR164, and expression of its target genes *CUC1* and *CUC2*. (B) miR168 accumulation, the unchanged *drb235* miRNA class representative and expression of its target gene *AGO1*. (C) Assessment of miRNA accumulation and precursor transcript and target gene expression for the reduced *drb235* miRNA class representative, miR169.

To determine whether the deregulated miRNA accumulation observed in the SAM region of the *drb235* triple mutant was a result of the loss of activity of one or multiple DRB proteins, mature miRNA accumulation as well as pri-miRNA and target gene expression was assessed in the same tissues used for sRNA sequencing sampled from all possible *drb2*, *drb3* and *drb5* mutant combinations. Northern blotting revealed a clear association between the loss of DRB2 activity (Figure S3A) and enhanced mature sRNA accumulation for the elevated miRNA class representative, miR164 (Figure 2A). RT-PCR analysis of pri-miRNA expression suggested that the enhanced accumulation of miR164 observed in the *drb2*, *drb23*, *drb25* and *drb235* mutant backgrounds resulted from more efficient processing of the precursor transcripts, *PRI-MIR164A* and *PRI-MIR164B* in the absence of DRB2 activity (Figure 2A). Furthermore, RT-PCR analysis revealed that in accordance with enhanced precursor transcript processing and mature miRNA accumulation, the expression levels of two of the targets of miR164, namely *CUC1* and *CUC2* were reduced. *CUC1* was undetectable in all *drb2*-containing plant lines. Intriguingly, *CUC2* was only undetectable in *drb235* plants. In *drb2*, *drb23* and *drb25* plants *CUC2* was detectable, but at significantly reduced levels. Taken together, these results suggest that these two closely related targets have different DRB requirements for regulation of their wild-type expression by miR164. The expression of *CUC3*, a closely related member of the same transcription factor family as *CUC1* and *CUC2* was also assessed by RT-PCR. Unlike *CUC1* and *CUC2*, *CUC3* does not contain a miR164 target sequence, however, *CUC3* expression has been shown to be regulated by these two closely related family members [33], [34]. The observed reduction in *CUC3* levels in all four of the analyzed *drb2*-containing backgrounds suggested that miR164 target gene expression was indeed reduced in the same tissues where miR164 levels were shown to be elevated by northern blotting.

MiR168 was selected as the unchanged miRNA class representative, as we have previously demonstrated that the accumulation of this miRNA is largely unaffected by the loss of activity of any of the five members of the *Arabidopsis* DRB protein family in whole plant samples [25], [35]. Northern blotting and RT-PCR analyses confirmed our previous findings, demonstrating that precursor transcript (*PRI-MIR168A*) processing efficiency, mature miRNA accumulation and target gene (*AGO1*) expression all remained at approximate wild-type levels in the absence of DRB2, DRB3 and DRB5 activity (Figures 2B and S3A). However, contrary to the reported independence of miR168 accumulation in the absence of DRB activity in whole plants, these analyses also revealed that in the specific tissue analyzed by sRNA sequencing, the SAM region, DRB1 is required for wild-type miR168 accumulation and target gene expression regulation (Figure 2B).

As demonstrated for the elevated class of miRNAs, accumulation of the *drb235* reduced miRNA class representative, miR169 was associated with the loss of DRB2 activity (Figure S3A). MiR169 levels were reduced in *drb2*, *drb23*, *drb25* and *drb235* plants (Figure 2C). The *Arabidopsis* miR169 family consists of 14 members and our sRNA sequencing revealed miR169a to be the most prevalent of the five family members detected (Table S1). The precursor transcript and target gene of miR169a, *PRI-MIR169A* and *NFYA5* respectively [36] were therefore included in our analyses. RT-PCR analysis suggested that the observed reductions in miR169 accumulation in *drb2*, *drb23*, *drb25* and *drb235* plants was a result of inefficient primary transcript processing with higher levels of *PRI-MIR169A* detected in all four of these DRB2 deficient backgrounds (Figure 2C). The reductions in mature miR169 accumulation due to inefficient *PRI-MIR169A* processing observed in *drb2*, *drb23*, *drb25* and *drb235* plants was in turn demonstrated to result in deregulated target gene expression with *NFYA5* levels elevated in all four of these plant lines lacking DRB2 activity (Figures 2C and S3A).

Northern blotting was further applied to confirm the association between the loss of DRB2 activity and alterations to miRNA accumulation for both the *drb235* elevated and reduced miRNA classes. As demonstrated for miR164 and miR169, Figure S3B shows that the levels of two additional miRNAs, specifically miR841 and miR170 (Table 1), are elevated and reduced respectively in the absence of *DRB2* expression (Figure S3A). Furthermore, and as illustrated for miR168, miR162 accumulation remained at wild-type levels in the absence of *DRB2*, *DRB3* and *DRB5* expression (Figure S3). Concurrent examination of these analyses strongly indicated that DRB2 activity is associated with the observed changes to miRNA accumulation for both the *drb235* elevated and reduced classes of miRNA, and that in these specific tissues DRB family members DRB2, DRB3 and DRB5 are not involved in the biogenesis of miRNAs exhibiting wild-type levels of accumulation.

### The *drb235* phenotype results from the tissue-specific elevation of miR164 accumulation

**Figure 3:**
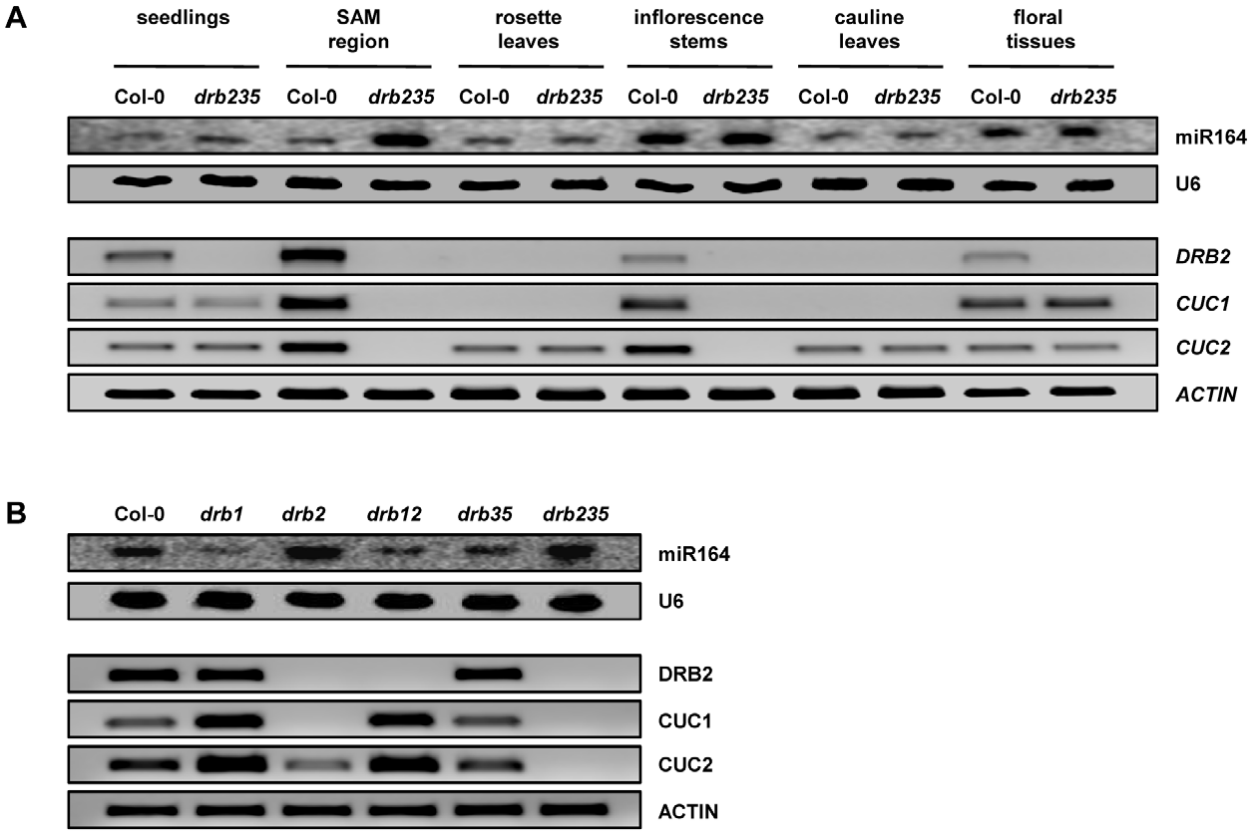
The tissue-specific over-expression of miR164. (A) Northern blot assessment of miR164 accumulation and RT-PCR analysis of *DRB2*, *CUC1* and *CUC2* expression in specific tissues collected from Col-0 and *drb235* plants. (B) Plants defective for DRB1 and DRB2 activity express distinct phenotypes and show differential miR164 accumulation and target gene expression in the SAM region.

The *drb235* developmental phenotype is primarily characterized by rosette leaf margin serration and inflorescence stem fusion (Figures 1A and 1B). Changes to miR164 accumulation and/or the expression of two of its target genes, *CUC1* and *CUC2* are associated with alterations to rosette leaf margin serration as well as defects in SAM formation and cotyledon, sepal and stamen separation [31], [37], [38]. MiR164 accumulation, along with *DRB2*, *CUC1* and *CUC2* expression was therefore assessed in specific tissues of Col-0 and *drb235* plants to correlate these expression changes with the *drb235* phenotype. Northern blotting showed that in wild-type plants, miR164 levels are spatiotemporally regulated. The miR164 sRNA accumulated to detectable levels in all Col-0 tissues evaluated, with the highest levels of accumulation detected in the inflorescence stem and floral tissue (Figure 3A). RT-PCR analysis revealed that *DRB2*, together with the miR164 targets *CUC1* and *CUC2* showed high levels of overlapping expression in the Col-0 SAM region sample (Figure 3A). Northern blotting showed that miR164 accumulation was highly elevated in the corresponding tissue of *drb235* plants and in accordance RT-PCR revealed a corresponding loss of target gene expression. A similar trend was also observed for the *drb235* inflorescence stem sample where the loss of DRB2 activity was demonstrated to result in enhanced miR164 accumulation and a corresponding loss of *CUC1* and *CUC2* expression (Figure 3A).

These analyses also revealed that enhanced mature miR164 accumulation in the absence of *DRB2* expression is tissue-specific. Although *DRB2* was expressed at low levels in Col-0 seedlings and floral tissues, no change in miR164 accumulation or target gene expression was observed in the corresponding tissue of *drb235* plants. Detection of *DRB2* expression in Col-0 seedlings was not unexpected as the analyzed tissue would also contain the SAM region where promoter-GUS (Figure 1C) and RT-PCR analyses (Figure 3A) showed *DRB2* expression to be localized. This suggests that other tissues collected as part of the seedling sample, including cotyledons, young leaves and roots are masking the observed changes in miR164, *CUC1* and *CUC2* levels in the SAM region of *drb235* seedlings. Plants lacking miR164c accumulation or ectopically expressing either the *MIR164A* or *MIR164B* precursor transcript produce abnormal floral organs as a consequence of altered CUC1 and CUC2 activities [30], [31], [39]. No floral defects are observed in *drb235* plants and furthermore no change in miR164c level was detected by sRNA sequencing (Table S1). This suggests that in wild-type plants, DRB2 does not interact with the *PRI-MIR164C* transcript and that the tissue-specific elevation of miR164 accumulation observed in plants lacking the activity of DRB2 results from a loss of the repressive effects of DRB2 on DCL1/DRB1-mediated, *PRI-MIR164A* and *PRI-MIR164B* processing (Figure 2A).

To further test the association between tissue-specific elevation of miR164 accumulation with the *drb235* developmental phenotype, miR164, *CUC1* and *CUC2* levels were assessed in additional *drb* mutants that also have altered miR164 accumulation but do not express the rosette leaf margin serration or inflorescence stem fusion defects of *drb235* plants. In addition to Col-0, the *drb35* double mutant was also included in these analyses as a wild-type control for miR164 accumulation, target gene expression (Figure 2A), leaf margin serration and inflorescence stem architecture (Figures 1A and 1B). We and others have previously shown that DRB1 is required for the biogenesis and wild-type accumulation of the miR164 sRNA [7], [40]. The analyses presented in Figure 3B show that in the SAM region of *drb1* plants, miR164 accumulation is significantly reduced and that the expression of its target genes, *CUC1* and *CUC2* is proportionately elevated. MiR164 accumulation and target gene expression are also reduced and up-regulated respectively in *drb12* plants, however these changes are not as severe as those detected in *drb1* plants. These four plant lines with either wild-type miR164 and target gene levels (Figure S4A; Col-0 and *drb35*), or reduced miR164 accumulation and up-regulated target gene expression (Figure S4B; *drb1* and *drb12*), all develop rosette leaves with smooth margins and inflorescence stems that are not fused.

Unlike *drb1* and *drb12* plants, altered miR164 accumulation correlates with the observed changes to rosette leaf margin serration and/or inflorescence stem architecture in *drb2* and *drb235* plants. Elevated miR164 accumulation in the SAM region of *drb2* plants leads to the loss of *CUC1* expression and significantly reduced levels of *CUC2* (Figure 3B). In the same tissues in the *drb235* triple mutant however, enhanced miR164 accumulation results in the complete loss of both *CUC1* and *CUC2* expression. The additional loss of *CUC2* expression in *drb235* plants, compared to the loss of *CUC1* only in *drb2*, appears to direct the differences in inflorescence stem architecture displayed by these two DRB2-defective plant lines (Figures 1B and S4C). These analyses also suggest that the observed reductions to *CUC2* expression and the complete loss of *CUC1* are responsible for the development of rosette leaf margin serration in all *drb2*-containing backgrounds.

### DRB1 and DRB2 are required for miRNA biogenesis in the SAM region

**Figure 4:**
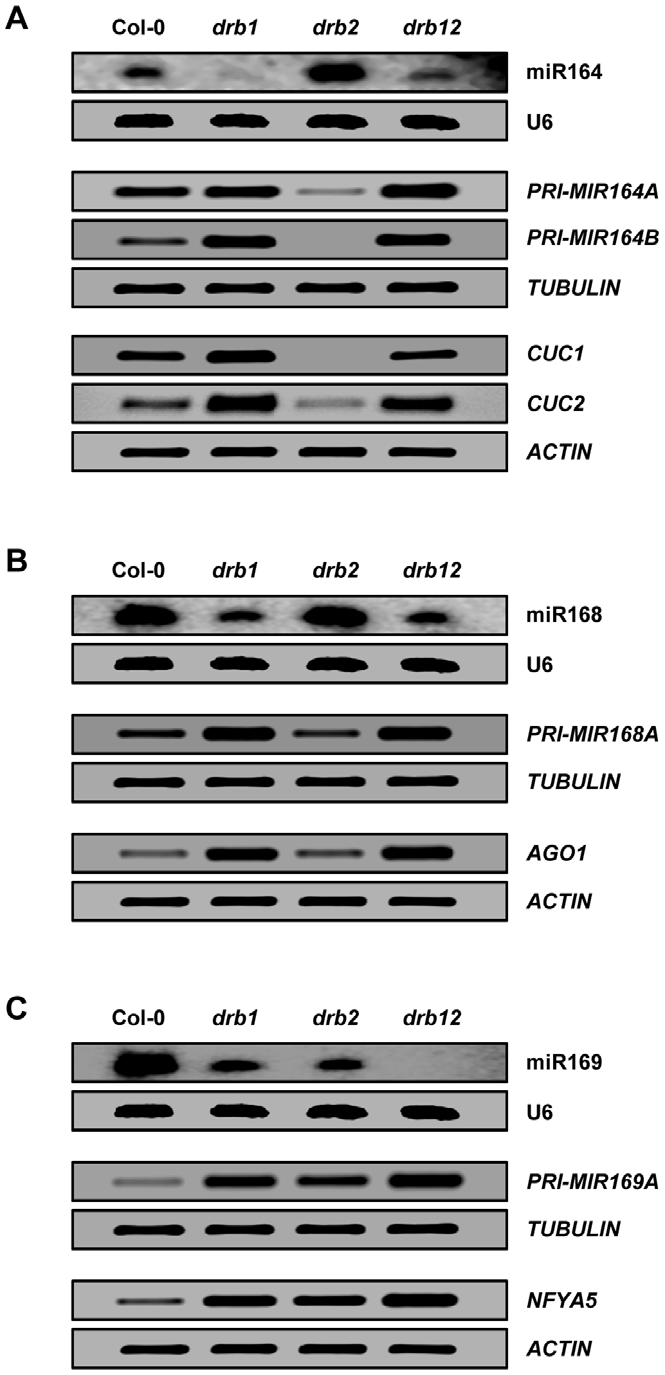
DRB1 and DRB2 are required for miRNA biogenesis in the SAM region. (A) miR164 accumulation, *MIR164* precursor transcript processing and target gene expression in the SAM region of *drb1*, *drb2* and *drb12* plants. (B) miR168 accumulation and *PRI-MIR168A* and *AGO1* expression in *drb1*, *drb2* and *drb12*. (C) The accumulation of miR169 and the expression of *PRI-MIR169A* and *NFYA5* in *drb1*, *drb2* and *drb12* plants.

To determine the contribution of DRB1 and DRB2 activity to miRNA biogenesis in the SAM region northern blotting and RT-PCR were used to assess miRNA accumulation, precursor transcript processing and target gene expression for the *drb235* elevated, unchanged and reduced miRNA class representatives in *drb1*, *drb2* and *drb12* plants. Figure 4A shows that compared to *drb1* and *drb2* plants, miR164 accumulation is elevated and reduced, respectively, in the *drb12* double mutant. RT-PCR revealed a direct correlation between precursor transcript processing efficiency, *PRI-MIR164A* and *PRI-MIR164B* expression, and miRNA accumulation in these three *drb* mutant lines. Target gene expression was also reflective of precursor transcript processing efficiency and mature miRNA accumulation. The data presented in Figure 4A suggests that in the absence of DRB2 activity in *drb12* plants, miR164 precursor transcripts are more freely available to enter the canonical miRNA biogenesis pathway mediated by the DCL1/DRB1 partnership, but in this double mutant plant DCL1 cannot efficiently process the increased levels of available substrate as it is also defective in DRB1 activity.

Compared to wild-type plants, no change in the levels of miR168, the precursor transcript *PRI-MIR168A* or the target gene *AGO1* were observed in *drb2* plants (Figure 4B). Changes in precursor transcript processing efficiency, mature miRNA accumulation and target gene expression were observed in *drb1* and *drb12*. However, the molecular profile of the *drb12* double mutant exactly matched that of *drb1* plants demonstrating that DRB2 is not involved in the biogenesis of miRNAs with unchanged accumulation in the SAM region of *drb235* plants (Table S1).

The accumulation of the *drb235* reduced miRNA class representative, miR169 was reduced in all three *drb* mutant backgrounds analyzed. Compared to Col-0, miR169 was at reduced yet detectable levels in *drb1* and *drb2* plants, but below detection sensitivities in the *drb12* double mutant (Figure 4C). RT-PCR assessment of *PRI-MIR169A* expression showed that the observed reduction to miR169 accumulation in *drb1* and *drb2* plants was a result of inefficient precursor transcript processing. The detection of even higher levels of precursor transcript, in combination with the failure to detect miR169 by northern blotting, in *drb12* plants strongly indicated that the activity of both DRB family members is a requirement for miRNA biogenesis in the SAM region of *Arabidopsis* plants.

### An artificial miRNA directing PHYTOENE DESATURASE silencing is differentially processed from individual miRNA precursor transcripts

**Figure 5:**
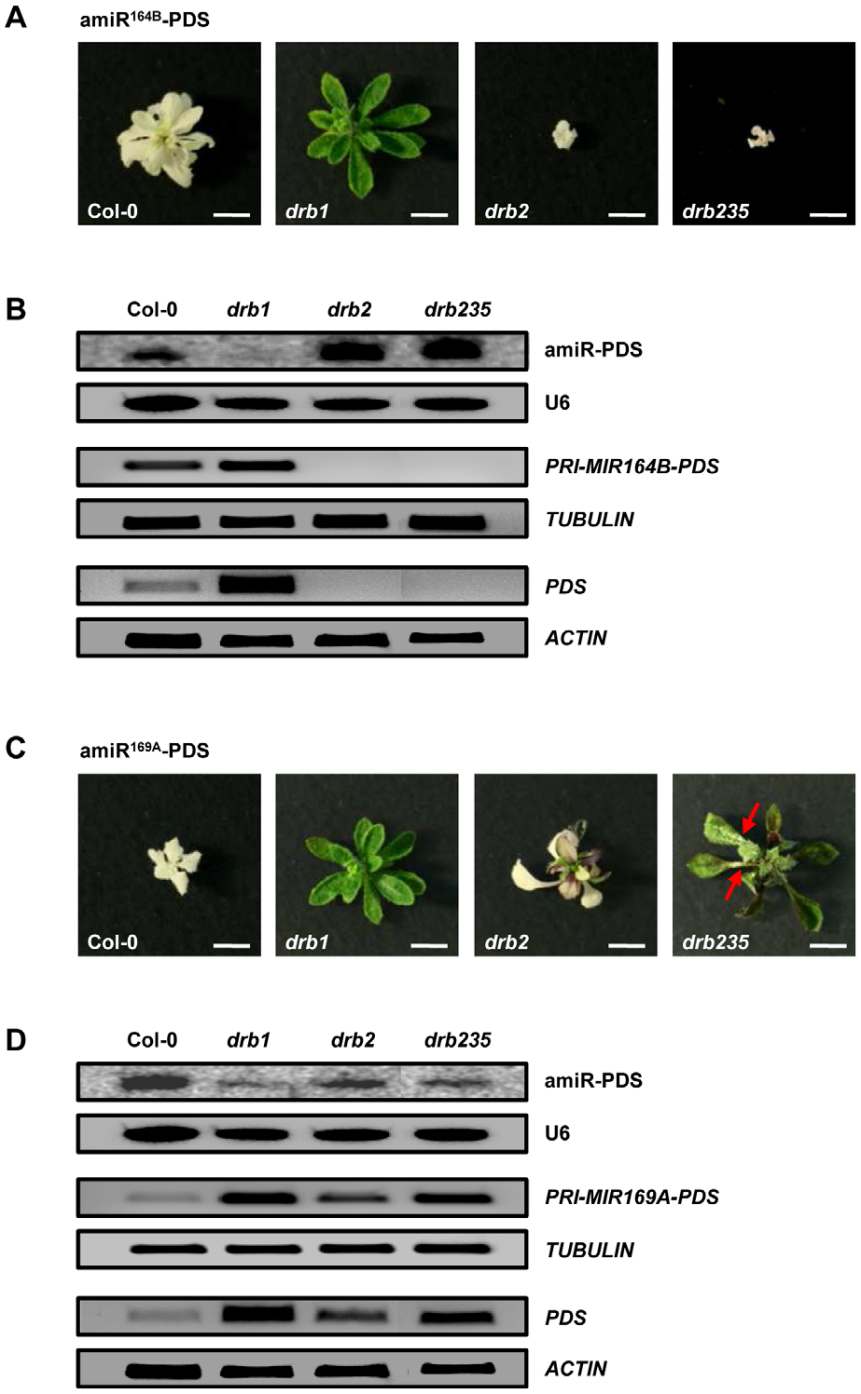
Artificial miRNA-directed silencing of *PDS*. (A) Photo-bleached phenotypes expressed by Col-0 and *drb* mutants transformed with the amiR^164B^-PDS plant expression vector. Scale bars = 7.5 mm. (B) amiR-PDS accumulation and *PRI-MIR164B-PDS* and *PDS* expression in amiR^164B^-PDS expressing plants. (C) amiR^169A^-PDS-directed PDS silencing in wild-type plants and *drb* mutants. Red arrows indicate the small sectors of photo-bleaching displayed by *drb235*/amiR^169A^-PDS plants. Scale bars = 7.5 mm. (D) amiR-PDS accumulation and target transcript expression in plants expressing the modified *PRI-MIR169A* transcript targeting *PDS* for amiRNA-directed silencing.

The results presented in Figure 4 indicated that the involvement of DRB1 and DRB2 in miRNA biogenesis in the SAM region of *Arabidopsis* plants is determined at the miRNA precursor transcript level. To further assess the influence of the miRNA precursor transcript on DRB1- and DRB2-mediated miRNA-directed silencing, the endogenous miRNA sequences of *PRI-MIR164B* and *PRI-MIR169A* were replaced with an identical artificial miRNA (amiRNA) sequence targeting *PHYTOENE DESATURASE* (*PDS*; amiR-PDS) for amiRNA-directed RNA silencing. These two plant expression vectors, amiR^164B^-PDS and amiR^169A^-PDS, were generated via overlapping PCR-based cloning [41], [42] and used to transform wild-type Col-0 plants and *drb* mutants, *drb1*, *drb2* and *drb235*. Transformation of Col-0 with either PDS-targeting amiRNA vector generated plants with rosette leaves that were completely photo-bleached (Figures 5A and 5C). Both amiR-PDS vectors were also introduced into *drb1* plants that lack the activity of DRB1, the preferred partner protein of DCL1 in miRNA biogenesis. Unlike Col-0, introduction of either amiRNA vector into *drb1* plants resulted in the generation of transformant lines that were essentially *drb1* in appearance, lacking any observable photo-bleaching or further arrest of *drb1* development. Northern blot and RT-PCR analyses of amiR-PDS accumulation, precursor transcript processing and target transcript expression demonstrated that the differences in silencing efficiencies directed by either amiRNA vector in Col-0 and *drb1* plants was a result of inefficient precursor transcript processing in the absence of DRB1 activity, leading to reduced mature amiR-PDS accumulation and defective silencing of the *PDS* target gene in *drb1* transformant lines (Figures 5B and 5D).

The photo-bleached phenotype of Col-0/amiR^164B^-PDS plants was uniformly expressed by *drb2* and *drb235* plants following transformation with the amiR^164B^-PDS vector (Figure 5A). However, molecular analyses, as demonstrated by northern blotting and RT-PCR, showed that the over-accumulation of the *PRI-MIR164B*-delivered sRNA in the absence of DRB2 activity, resulted in additional severe reductions to overall plant growth and development in *drb2*/amiR^164B^-PDS and *drb235*/amiR^164B^-PDS plants. All recovered *drb2*/amiR^164B^-PDS and *drb235*/amiR^164B^-PDS transformants were further reduced in size compared to Col-0/amiR^164B^-PDS transformants (Figure 5A). Taken together, these results again associated the loss of DRB2 activity with enhanced *PRI-MIR164B* processing, mature miR164 (amiR-PDS) accumulation and sRNA-directed target gene (*PDS*) silencing (Figure 5B).

As reported for Col-0/amiR^169A^-PDS plants, the cotyledons and first few leaf pairs of amiR^169A^-PDS-transformed *drb2* plants were completely photo-bleached. However, as these transformants matured, rosette leaves with green tissue emerged from the SAM region (Figure 5C). Interestingly, this is the same tissue where reporter gene expression was observed in *DRB2* promoter-driven GUS lines (Figure 1C). In accordance with the emergence of green tissue, amiR-PDS accumulation was reduced in *drb2*/amiR^169A^-PDS plants, and furthermore, precursor transcript processing and target gene expression were determined to be reduced and elevated respectively in the *drb2* background (Figure 5D). As described for *drb1*/amiR^169A^-PDS transformants, *drb235* plants expressing the amiR^169A^-PDS vector closely resembled the appearance of the parental line. However, small sectors of photo-bleaching were occasionally observed in rosette leaves, rosette leaf petioles (Figure 5C; red arrows) and the inflorescence stem of *drb235*/amiR^169A^-PDS transformants. Accumulation of the amiR-PDS sRNA was reduced in *drb235* transformant lines and RT-PCR suggested that this reduction resulted from inefficient *PRI-MIR169A-PDS* processing in the triple mutant, leading to deregulated target gene expression (Figure 5D). When compared with amiR^169A^-PDS transformed *drb2* plants, the almost complete lack of photo-bleaching in *drb235*/amiR^169A^-PDS transformants strongly indicates that the action of all three of these closely-related DRB family members is required for wild-type sRNA-mediated target gene expression regulation for miRNAs with reduced accumulation in *drb235* plants.

### The constitutive over-expression of DRB2 can compensate for the loss of DRB1

**Figure 6:**
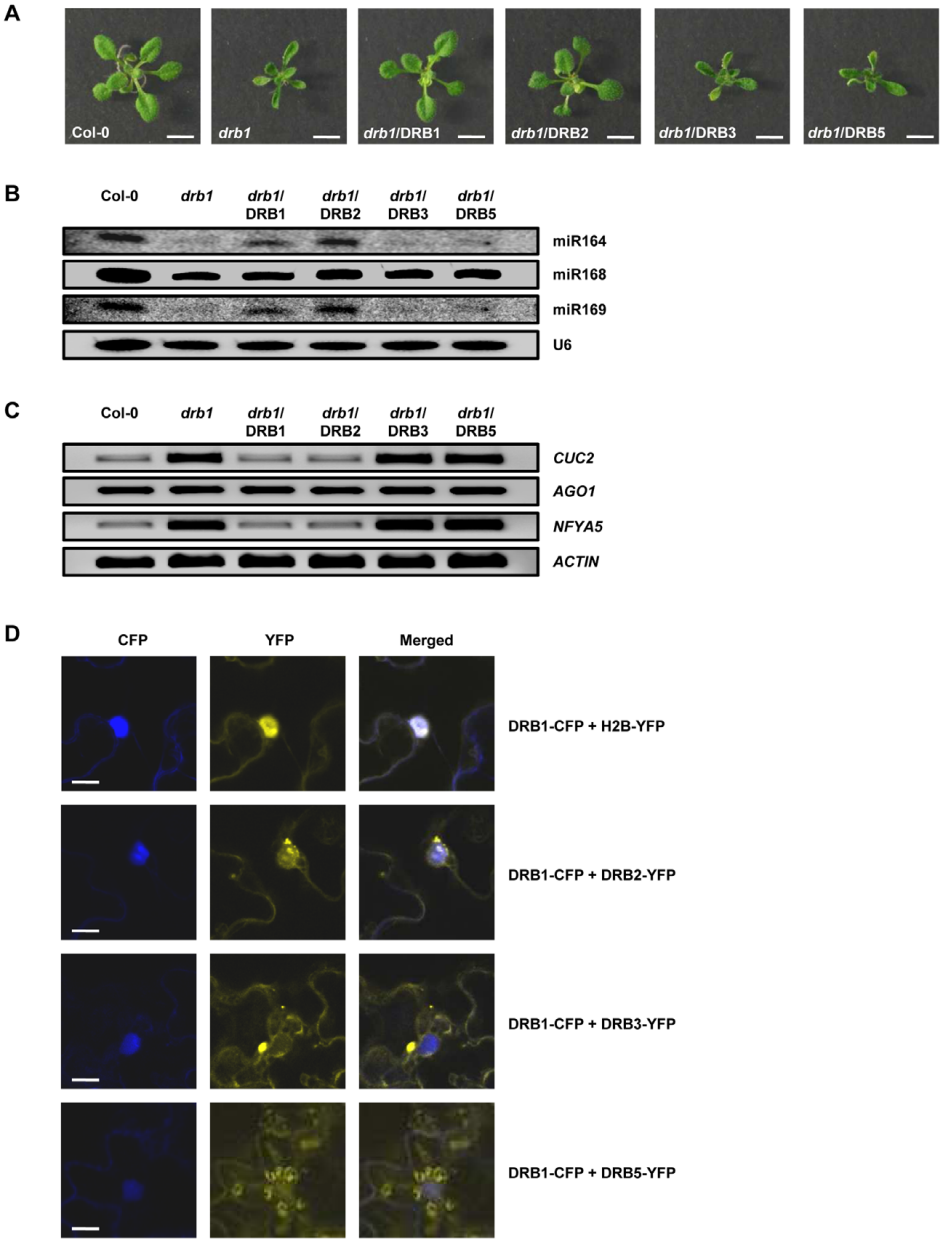
The constitutive over-expression of DRB2 can compensate for the loss of DRB1 activity. (A) Phenotypes expressed by homozygous 2 week old plants following the transformation of *drb1* with the DRB1, DRB2, DRB3 and DRB5 over-expression vectors. Scale bars = 7.5 mm. (B) miR164, miR168 and miR169 accumulation in DRB over-expression vector transformed *drb1* whole plants. (C) *CUC2*, *AGO1* and *NFYA5* expression, the target genes of miR164, miR168 and miR169 respectively in DRB over-expression vector transformed *drb1* whole plants. (D) Cellular localisation of *Arabidopsis* DRB1, DRB2, DRB3 and DRB5 fluorescent reporter gene fusion vectors transiently expressed in *N. benthamiana* leaves. Scale bars = 20 µm.

To confirm the requirement of DRB2 activity in miRNA biogenesis, *drb1* plants were transformed with the *DRB2* coding sequence under the control of the 35S promoter (Figure S5A). We have previously shown that the *drb1* developmental phenotype can be fully complemented via the introduction of a plant expression vector that constitutively over-expresses *DRB1* [25]. This transformant line was therefore included in our analyses as a positive control for reversion to wild-type miRNA accumulation and target gene expression. Similarly to the positive control line *drb1*/DRB1, the constitutive over-expression of *DRB2* (Figure S5B), in the absence of DRB1 activity, fully complemented the *drb1* phenotype (Figure 6A). Northern blotting (Figure 6B) and RT-PCR (Figure 6C) analyses further demonstrated that the molecular profile of *drb1*/DRB2 plants closely matched those of Col-0 and *drb1*/DRB1 plants. The coding sequences of *DRB3* and *DRB5* were also constitutively expressed in the *drb1* background to determine if these two DRB family members could also compensate for the loss of DRB1 activity (Figures S5A and S5B). However, no transformant line expressing a wild-type phenotype was recovered following transformation of *drb1* plants with either vector (Figure 6A). In addition to displaying the *drb1* phenotype, mature miRNA accumulation and target gene expression remained at *drb1* levels in *drb1*/DRB3 and *drb1*/DRB5 plants (Figures 6B and 6C).

To determine why the constitutive over-expression of DRB2, and not DRB3 or DRB5 allowed for *drb1* complementation, the coding sequences of *DRB2*, *DRB3* and *DRB5* were fused in frame with the *YELLOW FLOURESCENT PROTEIN* (*YFP*) reporter gene and transiently expressed in *Nicotinia benthamiana* (*N. benthamiana*) leaves via *Agrobacterium*-infiltration. Previous studies have shown DRB1, DCL1 and their miRNA precursor substrates to be localized in nuclear D-bodies [14], [15], [16]. The *DRB1* coding sequence was therefore fused to the *CYAN FLOURESCENT PROTEIN* (*CFP*) reporter gene for; i) co-expression with the DRB2-YFP, DRB3-YFP and DRB5-YFP vectors, and; ii) confirmation of nuclear localization. As an additional positive control for nuclear localization, we used the Histone 2B (H2B) protein fused in frame to YFP (H2B-YFP) which has been demonstrated previously to exclusively localize YFP florescence to the nucleus [43]. As expected, co-infiltration of *N. benthamiana* leaves with the DRB1-CFP and H2B-YFP constructs showed overlapping expression in the nucleus of infiltrated cells (Figure 6D). Reporter gene expression also overlapped in the nucleus when the DRB1-CFP and DRB2-YFP vectors were co-infiltrated with YFP fluorescence concentrated in small nuclear compartments and adjacent to the nuclear membrane. However, YFP and CFP fluorescence did not overlap when either the DRB3-YFP or DRB5-YFP vector was co-expressed with DRB1-CFP. YFP was observed throughout the cytoplasm of cells expressing DRB3-YFP. Fluorescence was also observed in the cytoplasm of DRB5-YFP expressing cells, concentrating in chloroplasts (Figure 6D). Taken together, the results presented in Figure 6 suggest that DRB3 and DRB5 are not able to substitute for DRB1 activity in the DCL1-catalyzed dsRNA processing stages of the *Arabidopsis* miRNA biogenesis pathway as these two family members are excluded from the appropriate cellular compartment, namely nuclear D-bodies. Furthermore, these analyses also demonstrated that when expressed constitutively outside of its wild-type functional domain, nuclear-localized DRB2 can compensate for the loss of DRB1 activity in *drb1* plants.

## Discussion

In this study, we have demonstrated that the pleiotropic phenotype displayed by *drb235* plants is a result of altered miRNA accumulation and target gene expression in specific tissues where *DRB2* is expressed in wild-type plants. Extension of our original *DRB2* putative promoter region from 1.7 kb [25] to 4 kb upstream of the DRB2 transcription start site showed that these additional regulatory elements directed *DRB2* expression to overlap with *DRB3* and *DRB5* in the SAM region (Figure 1C). The *drb235* developmental phenotype when compared to those displayed by *dcl1* hypomorphic and *drb1* null mutants suggested that DRB2, DRB3 and DRB5 could be functioning redundantly in the *Arabidopsis* miRNA biogenesis pathway. Small RNA sequencing of the specific tissue where these three genes are expressed in wild-type plants identified three distinct miRNA accumulation classes in *drb235* plants, those that were elevated, unchanged or reduced. Northern blotting (Figures 2 and S3) revealed a clear association between the loss of DRB2 activity and altered sRNA levels for both the elevated and reduced *drb235* miRNA classes to suggest that DRB2 under some circumstances is antagonistic and under other circumstances is synergistic to the function of DRB1 in miRNA biogenesis in this developmentally important tissue.

The accumulation of the elevated miRNA class representative, miR164, was enhanced in all plant lines lacking DRB2 activity (Figures 2A and S3A). However, the phenotype of the *drb235* triple mutant is distinct to *drb2*, *drb23* and *drb25* and all other *drb* mutant combinations, developing fused inflorescence stems (Figure 1B). In this tissue (Figure 3A), and in the SAM region of *drb235* plants (Figures 2A and 3A), the elevated levels of miR164 completely represses *CUC1* and *CUC2* expression. Previous genetic analyses have shown that CUC1 and CUC2 are functionally redundant and that plants defective for CUC1 and CUC2 activity, including the *cuc1 cuc2* double mutant, or plants engineered to constitutively and ubiquitously express either the *MIR164A* or *MIR164B* precursor transcript, display vegetative and floral organ fusion defects [30], [33], [37], [38], [44]. This indicates that the inflorescence stem fusions observed in *drb235* plants result from tissue-specific elevation of miR164 accumulation and a corresponding loss of *CUC1* and *CUC2* expression.

Curiously, the phenotypic consequences of altered miR164, *CUC1* and *CUC2* levels on rosette leaf margin development reported here contrast with those described previously. The leaf margins of *cuc1-13* plants are indistinguishable from those of Col-0 plants and rosette leaves with smooth margins are displayed by the *cuc2-3* mutant or by plants ectopically over-expressing miR164 precursor transcripts [30], [31], [33]. In addition, T-DNA insertion knockouts of the *MIR164A* locus or plants engineered to express a miR164-resistant version of *CUC2* (CUC2g-m4 plants), develop highly serrated rosette leaves [31], [34], [45]. Taken together, these studies demonstrate that miR164a-mediated regulation of *CUC2* expression is required for rosette leaf margin development. In our series of *drb* mutants, including *drb2*, *drb23*, *drb25* and *drb235* plants, all of which were shown to have elevated miR164 levels and corresponding reductions or complete loss of *CUC1* and *CUC2* expression, developed rosette leaves with serrated margins (Figures 1A, 3B and S4). In contrast, miR164-mediated *CUC1* and *CUC2* expression is deregulated in *drb1* and *drb12* plants where miR164 levels are reduced and both of these mutant lines display rosette leaves with smooth margins (Figures 3B and S4B). Our analyses suggest that loss of *CUC1* expression and reduced *CUC2* levels in the specific tissues where miR164 accumulation is elevated in the absence of DRB2 activity, namely the SAM region, directs the rosette leaf margin serration phenotype expressed by *drb2*, *drb23*, *drb25* and *drb235* plants. These tissue-specific alterations to miR164 accumulation and target transcript expression could account for the phenotypic differences displayed by our *drb* mutant lines and those previously characterized for plants lines where miR164, *CUC1* and *CUC2* levels are altered in all tissues and throughout all stages of development.

RT-PCR analysis of *drb1*, *drb2* and *drb12* plants revealed that the observed changes to miR164 and miR169 levels in these three mutant lines was a result of alterations to precursor transcript processing efficiency (Figure 4). *PRI-MIR164A* and *PRI-MIR164B* expression was reduced in *drb2*, but the level of each precursor transcript was increased in *drb1* and *drb12* plants. The moderate increase in *PRI-MIR164A* and *PRI-MIR164B* levels in *drb12* plants compared to their higher levels of expression in *drb1* plants correlated with elevated miR164 accumulation in the double mutant (Figure 4A). These analyses indicate that DRB2 antagonism of the DCL1/DRB1 partnership during the precursor transcript processing stage of miRNA biogenesis is required to regulate the accumulation of a subset of miRNAs in the SAM region of wild-type *Arabidopsis* plants. Compared to wild-type plants, miR169 accumulation was reduced to a similar level in *drb1* and *drb2* (Figure 4C). In accordance with miR169 levels, *PRI-MIR169A* expression was elevated in both of these *drb* mutant plants to suggest that DRB1 and DRB2 are required for DCL1-catalyzed processing of the precursor transcripts of *MIR169* family members. Failure to detect a miR169 signal by northern blotting and detection of further elevated *PRI-MIR169A* and *NFYA5* expression in the *drb12* double mutant by RT-PCR confirmed that DRB2 activity in addition to DRB1 function is required for the biogenesis of a subset of miRNAs in the SAM region of *Arabidopsis* plants.

Modification of *PRI-MIR164B* and *PRI-MIR169A* to replace their endogenous sRNA silencing signals with the same *PDS*-targeting amiRNA confirmed that the antagonistic and synergistic action of DRB2 on DRB1 function in miRNA biogenesis occurs at the pri-miRNA level. Compared to Col-0 and *drb1* plants, transformation of *drb2* and *drb235* with the amiR^164B^-PDS vector resulted in further arrests to plant development due to an over accumulation of the amiR-PDS (Figures 5A and 5B). A different *PDS* silencing profile was displayed by the same *drb* mutants when expressing the second amiR-PDS vector. As shown for *drb1*/amiR^169A^-PDS plants, *PDS* silencing was severely deregulated in *drb235* plants expressing the amiR^169A^-PDS vector. AmiR^169A^-PDS-directed *PDS* silencing was also disrupted in *drb2* plants, but only in the specific tissue where DRB2 is expressed in wild-type plants (Figures 1C and 5C). Northern blotting and RT-PCR analyses directly correlated the efficiency of *PDS* silencing directed by either amiR-PDS vector with pri-miRNA processing efficiency and amiRNA accumulation in *drb2* and *drb235* plants.

Over-expressing *DRB2* in the absence of DRB1 activity fully complemented the *drb1* phenotype (Figure 6A). The wild-type miRNA accumulation and target gene expression profile of *drb1*/DRB2 whole plant samples (Figures 6B and 6C) suggests that the involvement of DRB2 in miRNA biogenesis is restricted by its tissue-specific expression (Figures 1C and 3A). The expression of individual *MIR* gene family members is also regulated both spatially and temporally [31], [32], [46], and although many of these pri-miRNA transcripts could potentially express the same structural features or sequence motifs as those demonstrated to require DRB2 for their biogenesis here, their wild-type accumulation in other tissues is only dependent on the ubiquitously expressed DRB1. Fusion of the DRB2 coding sequence to the YFP reporter gene showed that DRB2 is a nuclear protein (Figure 6D). DCL1 and DRB1 are also nuclear-localized and function in concert in D-bodies to direct cleavage of miRNA/miRNA* duplexes in a sequential two-step process from the dsRNA stem-loop regions of pri-miRNA and pre-miRNA transcripts [14], [16]. Furthermore, like DRB1, DRB2 has been demonstrated to interact with DCL1 and dsRNA *in vitro* [8] providing further support for the requirement of DRB2 activity in the biogenesis of a subset of miRNAs in specific tissues. It has recently been reported that DRB2 is antagonistic to DRB4 in the production of all siRNA size classes processed from PolIV generated transcripts and that DRB2 is synergistic to DRB4 in the biogenesis of DCL4-dependent miRNAs [26]. These findings parallel those reported here on DRB2 antagonism and synergism in DCL1/DRB1-directed miRNA biogenesis and suggest that the dsRNA intermediates derived from PolIV generated transcripts, or the stem-loop structures of DCL4/DRB4-dependent miRNA precursor transcripts also express structural features or sequence motifs that direct their interaction with DRB2 as well as with DRB4. Alternatively, DRB2 could be competing with DRB1 and DRB4 for interaction with their partnering proteins DCL1 and DCL4. Taken together, the results presented here and those of [26] suggest that DCL/DRB partnerships and/or DRB dsRNA interactions in the endogenous sRNA biogenesis pathways of *Arabidopsis* are more complex than previously thought.

As suggested by our northern blotting data (Figures 2C and 5D), DRB3 are DRB5 play no role in the processing steps of miRNA biogenesis and the constitutive and ubiquitous expression of these two DRB proteins failed to compensate for the loss of DRB1 activity in *drb1* plants (Figures 6A and 6B). YFP fusion to the DRB3 and DRB5 coding sequences revealed that they are not able to compensate for the loss of nuclear-localized DRB1 activity as they are both expressed in the cytoplasm (Figure 6D). However, these two cytoplasmic DRBs do appear to be involved in regulating the expression of specific target genes of DRB2-associated miRNAs. For example, the combined loss of DRB3 and DRB5 activity was demonstrated to result in the complete repression of *CUC2* expression in the SAM region of *drb235* plants (Figure 2A). Furthermore, the almost complete absence of photo-bleaching in *drb235*/amiR^169A^-PDS transformants compared to the tissue-specific loss of amiR^169A^-PDS-directed silencing only in *drb2*/amiR^169A^-PDS plants suggests that the activity of all three of these closely-related DRB family members is required for wild-type expression regulation of DRB2-associated miRNA target genes. The exact role that these two cytoplasmically-localized DRBs play in miRNA biogenesis or action remains to be functionally characterized.

**Figure 7:**
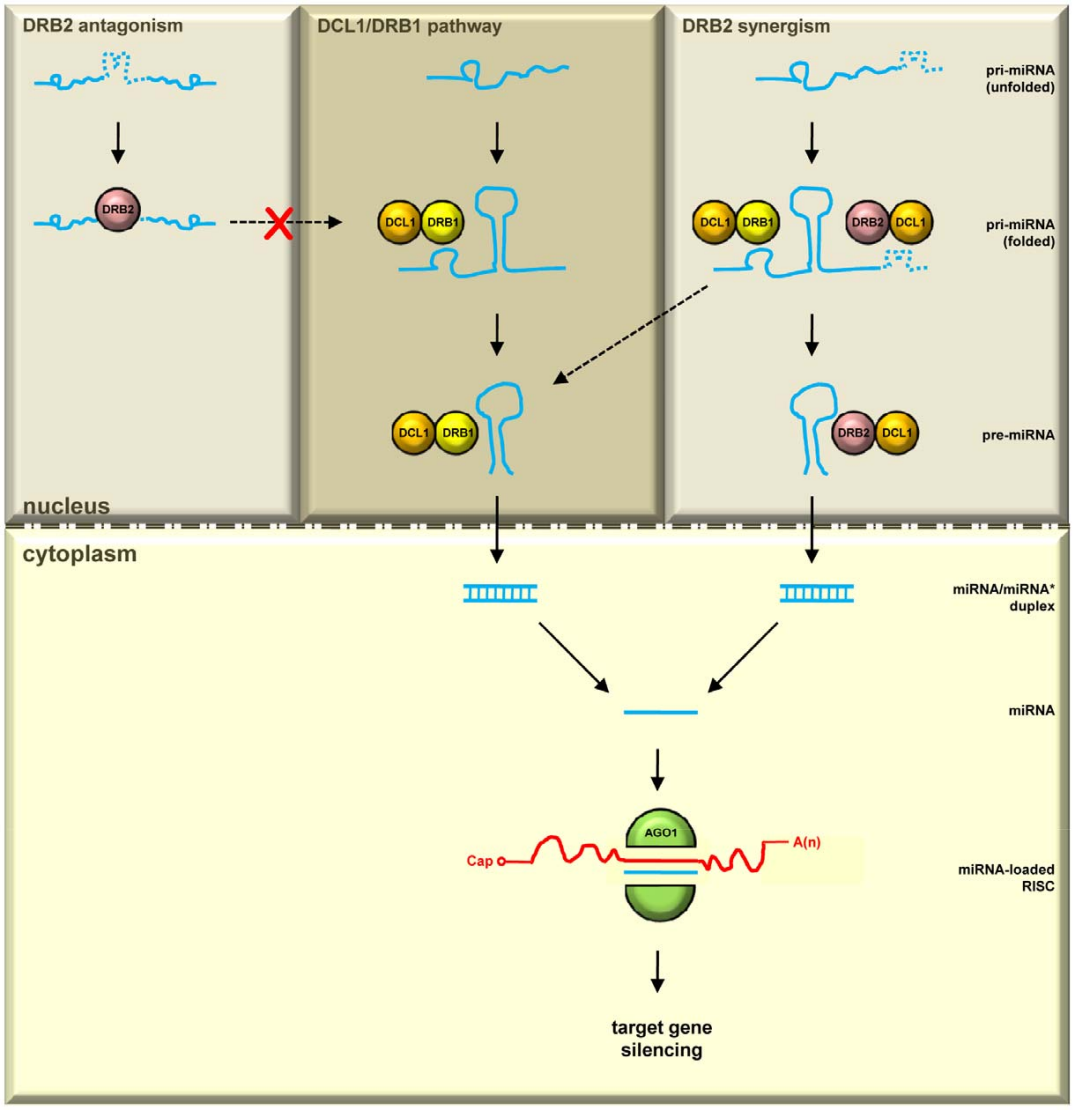
Proposed model for an alternate tissue-specific miRNA biogenesis pathway. In the SAM region, the established DCL1/DRB1 functional partnership is responsible for the biogenesis of miRNAs with unchanged accumulation in *drb235* plants (upper middle dark grey panel). For the *drb235* enhanced miRNA accumulation class, DRB2 is antagonistic to DRB1. We propose that these pri-miRNA transcripts express a DRB2-associating feature (dashed bumpy blue line) that prevents folding of the pre-miRNA stem-loop region and subsequent recognition and entry into the DCL1/DRB1 biogenesis pathway (upper left light grey panel). DRB2 is also synergistic to DRB1 for the biogenesis of miRNAs with reduced accumulation in *drb235* plants. These pri-miRNA transcripts are proposed to express features that direct their association with either DRB1 (solid bumpy blue line) or DRB2 (dashed bumpy blue line), allowing for their entry and processing by either the canonical DCL1/DRB1 partnership or the alternate tissue-specific DCL1/DRB2 partnership (upper right light grey panel).

We propose that, in *Arabidopsis*, DRB2 is performing a dual regulatory role in miRNA biogenesis in specific tissues (Figure 7). MiRNAs with wild-type accumulation in *drb235* plants are produced by the canonical miRNA biogenesis pathway mediated by the DCL1/DRB1 partnership (Figure 7; upper middle dark grey panel). Following their export to the cytoplasm the DCL1/DRB1-generated miRNA is loaded onto AGO1-catalyzed RISC to guide silencing of cognate mRNAs. For miRNAs with elevated accumulation in *drb235* plants our results suggest that their precursor transcripts harbor structural features or sequence motifs that direct their interaction with DRB2. For DRB2 to direct its antagonistic effect on the DCL1/DRB1 biogenesis pathway, the DRB2-associating signal is predicted to be positioned within the pri-miRNA sequence that would prevent pre-miRNA stem-loop formation. In the event that the DRB2-associating signal was positioned outside of this region, the pri-miRNA would still have the capacity to fold and be recognized for entry into the canonical miRNA biogenesis pathway (Figure 7; upper left light grey panel). DRB2 is also synergistic to DRB1 for the biogenesis of another distinct class of miRNAs. The precursor transcripts of miRNAs with reduced accumulation in *drb235* plants express associating signals that could direct their interaction with either DRB1 or DRB2 (Figure 7; upper right light grey panel). Directing different DRB/pri-miRNA transcript associations in tissues where *DRB2* is expressed would not only add an additional layer of regulatory complexity to miRNA-mediated target gene expression, but would also ensure the viability of plants in which either DRB1 or DRB2 activity is temporarily suppressed, for example by some environmental or pathogen-mediated stress.

## Materials and Methods

### Vector construction

The *DRB1*, *DRB3* and *DRB5* promoter driven *GUS* reporter gene plant expression vectors are previously described [25]. The new *DRB2* promoter-driven *GUS* reporter gene plant expression vector, p*DRB2*pro:GUS was generated by PCR amplification of a 4 kb genomic fragment upstream of the transcription start site of *DRB2* with primers pDRB2-PRO-F and pDRB2-PRO-R (Table S5). This PCR product was flanked by 5′ *Bam*HI and 3′ *Nco*I restriction sites and was cloned into the pGEM-T Easy cloning vector (Promega) to produce pGEM-T:*DRB2*pro. This vector was digested with *BamHI* and *NcoI* to release the *DRB2* promoter fragment that was cloned into the similarly digested vector pRITA [47] to produce pRITA:*DRB2*pro-GUS. The pRITA:*DRB2*pro-GUS vector was digested with *Not*I to release the *DRB2pro*:*GUS* fragment which was cloned into the similarly digested plant expression vector pBART [47] to produce p*DRB2*pro:GUS.

Genomic fragments containing the *PRI-MIR164B* (*AT5G01747*) and *PRI-MIR169A* (*AT3G13405*) sequences and flanking regulatory regions were amplified by PCR with primer pairs pMIR164B-F1/R1 and pMIR169A-F1/R1 and cloned into the pGEM-T Easy cloning vector to produce pGEM-T:*MIR164B* and pGEM-T:*MIR169A* respectively. These two vectors were used as templates for the construction of the *PDS*-targeting amiRNA plant expression vectors pamiR^164B^-PDS and pamiR^169A^-PDS by overlapping PCR-based cloning, essentially as described previously [41], [42]. The modified *PRI-MIR164B* and *PRI-MIR169A* sequences were cloned into pART7 using the introduced *Xho*I/*Eco*RI and *Xba*I/*Hind*III restriction sites. These two amiR-PDS shuttle vectors were digested with *Not*I and the resulting restriction fragments cloned into the similarly digested plant expression vector pBART to produce pamiR^164B^-PDS and pamiR^169A^-PDS. All primers used in the construction of the amiR-PDS vectors are listed in Table S5.

To produce the DRB over-expression vectors used to transform *drb1* plants, the *DRB1*, *DRB2*, *DRB3* and *DRB5* coding sequences were generated using the Qiagen OneStep RT-PCR kit according to the manufacturer's instructions (primer sequences listed in Table S5). Each product was flanked by a 5′ *Kpn*I and 3′ *Xma*I restriction site and these products cloned into the pGEM-T Easy cloning vector to produce vectors pGEM-T:DRB1/2/3/5. Vectors were digested with *Kpn*I and *Xma*I and the four resulting restriction fragments cloned into the similarly digested vector pART7 to produce pART7:DRB1/2/3/5. The pART7:DRB vector series was digested with *Not*I and cloned into the *Not*I site of pBART to produce the plant expression vectors pDRB1, pDRB2, pDRB3 and pDRB5.

For construction of the DRB fluorescent reporter gene fusion vector series, the DRB over-expression vectors were used as templates for PCR amplification of the respective DRB coding sequences, minus their stop codons. The antisense primer used in these PCR reactions introduced a *Xho*I restriction site at the 3′ terminus of each PCR product and these 4 no stop codon (NSC) DRB restriction fragments (DRB-NSC) were cloned into the pGEM-T Easy cloning vector to produce the pGEM-T:DRB1/2/3/5-NSC vectors. The *CFP* (DRB1) and *YFP* (DRB2, DRB3 and DRB5) reporter genes were also amplified by PCR with forward and reverse primers that contained *Xho*I restriction sites (Table S5). These two PCR products were cloned into the pGEM-T Easy cloning vector to produce pGEM-T:CFP and pGEM-T:YFP respectively and all vectors were subsequently digested with *Xho*I. The CFP/*Xho*I and YFP/*Xho*I restriction fragments were gel purified and cloned into the respective *Xho*I linearized pGEM-T:DRB1/2/3/5-NSC vectors. pGEM-T:DRB-NSC vectors containing reporter gene inserts in the desired 5′ to 3′ orientation were digested with *Eco*RI and cloned into the similarly digested pART7 to produce pART7:DRB1-CFP and pART7:DRB2/3/5-YFP respectively. These four vectors were digested with *Not*I and the resulting restriction fragments cloned into the *Not*I digested plant expression vector pBART to produce pDRB1-CFP, pDRB2-YFP, pDRB3-YFP and pDRB5-YFP.

### Plant material, growth conditions and transformations

The *drb* T-DNA insertion lines used in this study have been described previously [25]. *Arabidopsis* and *N. benthamiana* plants were grown under standard glasshouse conditions of 16 h of light/8 h of dark at 24°C. All plant expression vectors used in this study were transformed into *Agrobacterium tumefaciens* (strain GV3101) via electroporation. *Agrobacterium* cultures were used to transform *drb1* plants with the DRB series of over-expression vectors, wild-type plants (Col-0) with the *DRB2* promoter-driven *GUS* reporter gene vector and Col-0, *drb1*, *drb2* and *drb235* plants with the amiR-PDS vectors by floral dipping as described previously [48]. Transformants were selected by germinating the dipped seed on plant growth media supplemented with 10 mg/mL of phosphinothricin. For stable transformations of *Arabidopsis*, the number of primary transformants expressing the phenotypes reported here are listed in Table S6. Leaf infiltrations of *N. benthamiana* plants for the transient expression of the DRB fluorescent reporter gene fusion series of plant expression vectors via *Agrobacterium*-mediated transformation was performed as previously described [35].

### Reporter gene expression analyses

Screening for GUS expression in 4 week old plants expressing the *DRB1*, *DRB2*, *DRB3* or *DRB5* promoter-driven *GUS* reporter gene plant expression vectors using 5-bromo-4-chloro-3-indolyl-β-D-glucuronide (X-gluc) was conducted according to [49]. The *CFP* and *YFP* reporter gene vectors were transiently expressed in *Agrobacterium*-infiltrated *N. benthamiana* leaves for 72 hr. Following this incubation period, *Agrobacterium*-infiltrated leaves were removed from the plant and CFP and YFP fluorescence examined by confocal microscopy under constant illumination.

### Small RNA sequencing

Total RNA was isolated from the SAM region of 4 week old Col-0 and *drb235* plants using TRIzol Reagent according to the manufacturer's instructions (Invitrogen). Twenty micrograms (20 µg) of total RNA was shipped to the Victor Chang Cardiac Research Institute for processing. The sequences of sRNA (20–24 nt in length) that exactly matched those deposited into the miRBase database (http://www.mirbase.org/) for *Arabidopsis thaliana* were determined using SOLiD color space technology.

### Northern blot analysis of miRNA accumulation

Northern blot analysis to assess miRNA accumulation in *Arabidopsis* was essentially performed as described previously [40]. In brief, total RNA was isolated from pooled plant tissues using TRIzol Reagent according to the manufacturer's instructions. Twenty micrograms (20 µg) of total RNA was separated on 15% denaturing (10 M urea) polyacrylamide gels by electrophoresis and transferred to HyBond-N^+^ membrane (Amersham) by electroblotting. DNA oligonucleotide probes specific for each miRNA assessed by northern blotting were end-labeled using Terminal deoxynucleotidyl transferase (Fermentas) and α-^32^P CTP. All DNA oligonucleotide probes used in this study are listed in Table S4.

### RT-PCR and qRT-PCR analysis of pri-miRNA and miRNA target gene expression

A 5 µg aliquot of the same total RNA isolation used for northern blotting was digested with 5 units of RQ1 RNase-free DNase (Promega) at 37°C for 30 min and purified using an RNeasy Mini kit (Qiagen). Purified RNA (1 µg) was used to synthesize cDNA with SuperScript III reverse transcriptase (Invitrogen) and Oligo (dT)_23_ according to the manufacturer's instructions. Each cDNA was diluted to 50 ng/µL and 3 µL of this dilution was used as template in a 25 µL PCR reaction, and each experiment was repeated three times. *TUBULIN* (*TUBULIN BETA8*; *AT5G23860*) was used as the housekeeping control for pri-miRNA expression analysis and *ACTIN* (*ACTIN2*; *AT3G18780*) was used as the housekeeping control for miRNA target gene expression. All primers used in this study for RT-PCR assessment of pri-miRNA and miRNA target gene expression are listed in Table S5. To confirm RT-PCR assessment of precursor transcript and miRNA target gene expression, the same cDNA samples were analyzed by quantitative RT-PCR (qRT-PCR) according to [40]. All qRT-PCR expression data is provided in Table S7.

## Supporting Information

Figure S1 **Accumulation of miRNAs involved in leaf shape development and expression of their target genes in 4 week old whole plant samples.** (A) miR159, miR164 and miR319 accumulation in *drb* mutant whole plant samples. (B) *MYB33*, *CUC2* and *TCP4* expression, target genes of miR159, miR164 and miR319 respectively in *drb* mutant whole plant samples.
(TIF)

Figure S2 **miRNA accumulation in the SAM region of** ***drb235*** **plants.** (A) Accumulation of the 5 most up-regulated miRNAs, as determined by sRNA sequencing, in *drb235* plants. (B) Accumulation of 5 miRNAs determined to have unchanged levels by sRNA sequencing in *drb235* plants. (C) Accumulation of the 5 most down-regulated miRNAs, as determined by sRNA sequencing, in the SAM region of *drb235* plants.
(TIF)

Figure S3 **Comparison of miR841, miR162 and miR170 accumulation in the SAM region in** ***drb*** **mutant lines.** (A) RT-PCR confirmation of the absence of *DRB* expression in the SAM region samples collected from individual *drb* mutant lines and used for the molecular analyses presented in Figure 2. (B) Confirmation that the loss of DRB2 activity is associated with the observed alterations to mature miRNA accumulation for both the enhanced (miR841) and reduced (miR170) *drb235* miRNA accumulation class representatives.
(TIF)

Figure S4 **Leaf margin phenotypes displayed by** ***drb*** **mutants with altered miR164,** ***CUC1*** **and** ***CUC2*** **levels in the SAM region.** (A) Col-0 and *drb35* plants display rosette leaves with smooth margins and miR164, *CUC1* and *CUC2* levels are unchanged. (B) *drb1* and *drb12* plants have reduced miR164 accumulation and deregulated *CUC1* and *CUC2* expression and develop rosette leaves with smooth margins. (C) *drb2* and *drb235* plants develop rosette leaves with highly serrated margins and miR164 accumulation and target gene expression are elevated and reduced respectively in specific tissues of these mutant lines. (A to C) scale bars = 5 mm.
(TIF)

Figure S5 ***DRB*** **expression in** ***drb1*** **plants transformed with the DRB1, DRB2, DRB3 and DRB5 over-expression vectors.** (A) Schematic of the 35S promoter-driven plant expression vector for the over-expression of *DRB1*, *DRB2*, *DRB3* and *DRB5*. (B) RT-PCR analysis of *DRB* expression in *drb1* plants transformed with the DRB1, DRB2, DRB3 and DRB5 over-expression vectors. (C) Schematic of the 35S promoter-driven fluorescent reporter gene vectors developed to visualize the cellular locations of the *Arabidopsis* DRB1 (CFP), DRB2 (YFP), DRB3 (YFP) and DRB5 (YFP) proteins in *Agrobacterium*-infiltrated *N. benthamiana* leaves. (A and C) 35Sp, *Cauliflower mosaic virus* 35S promoter; DRB1/2/3/5 CDS, DRB coding sequences; NosT, nopaline synthase terminator.
(TIF)

Table S1 miRNA accumulation in the SAM region of *drb235* plants.
(DOC)

Table S2 *MIR* gene family accumulation in the SAM region of *drb235* plants.
(DOC)

Table S3 The *drb235* elevated, unchanged and reduced miRNA accumulation classes.
(DOC)

Table S4 DNA oligonucleotide probes used in this study.
(DOC)

Table S5 Primers used in this study.
(DOC)

Table S6 Phenotype expression in primary transformant lines generated in this study.
(DOC)

Table S7 qRT-PCR assessment of precursor transcript and miRNA target gene expression.
(DOC)
