# Supplementary figures and images for: DRB2 Is Required for MicroRNA Biogenesis in Arabidopsis thaliana
Source: PLoS One. 2012 Apr 24;7(4):e35933. doi: 10.1371/journal.pone.0035933 (PMC3335824; doi:10.1371/journal.pone.0035933)

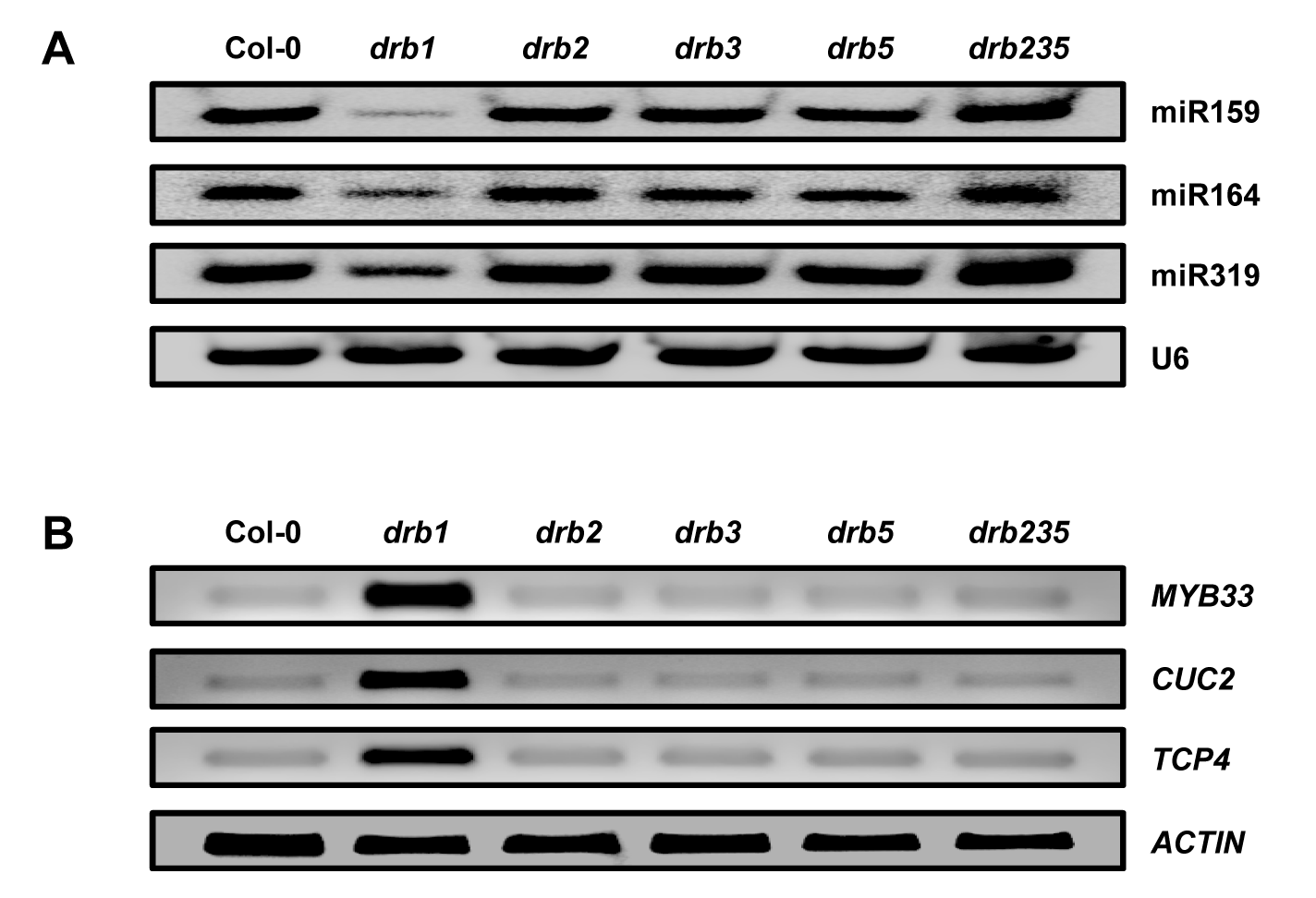

Supplement: Figure S1 — Accumulation of miRNAs involved in leaf shape development and expression of their target genes in 4 week old whole plant samples. (A) miR159, miR164 and miR319 accumulation in drb mutant whole plant samples. (B) MYB33, CUC2 and TCP4 expression, target genes of miR159, miR164 and miR319 respectively in drb mutant whole plant samples. (TIF) [file pone.0035933.s001.tif]

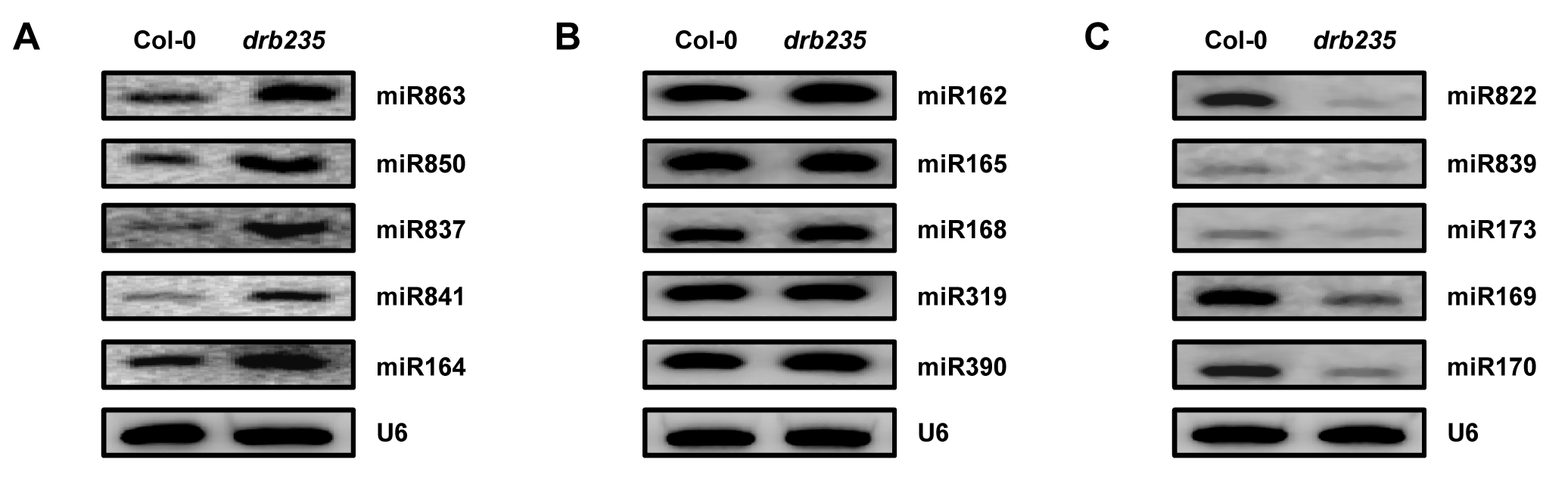

Supplement: Figure S2 — miRNA accumulation in the SAM region of drb235 plants. (A) Accumulation of the 5 most up-regulated miRNAs, as determined by sRNA sequencing, in drb235 plants. (B) Accumulation of 5 miRNAs determined to have unchanged levels by sRNA sequencing in drb235 plants. (C) Accumulation of the 5 most down-regulated miRNAs, as determined by sRNA sequencing, in the SAM region of drb235 plants. (TIF) [file pone.0035933.s002.tif]

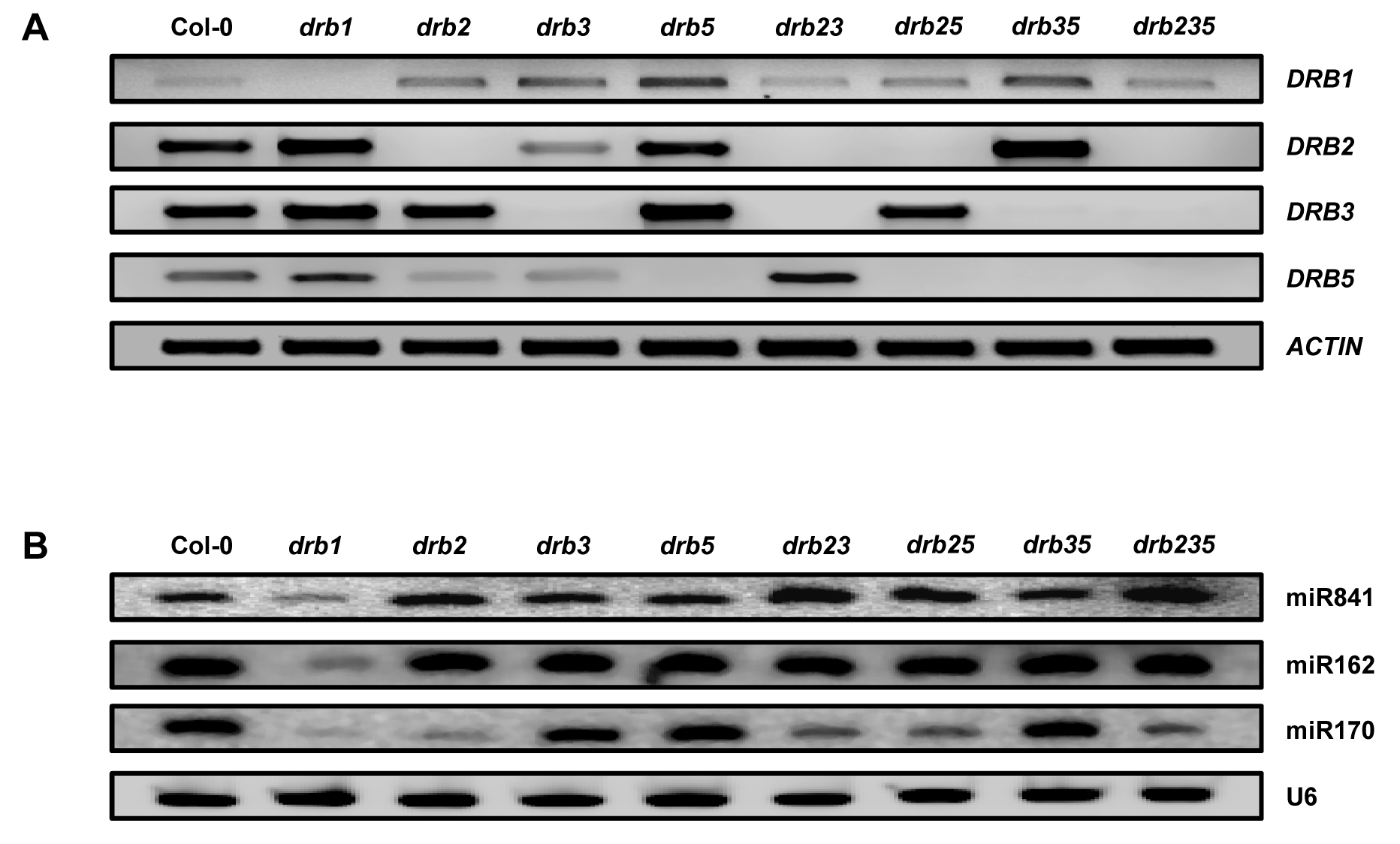

Supplement: Figure S3 — Comparison of miR841, miR162 and miR170 accumulation in the SAM region in drb mutant lines. (A) RT-PCR confirmation of the absence of DRB expression in the SAM region samples collected from individual drb mutant lines and used for the molecular analyses presented in Figure 2. (B) Confirmation that the loss of DRB2 activity is associated with the observed alterations to mature miRNA accumulation for both the enhanced (miR841) and reduced (miR170) drb235 miRNA accumulation class representatives. (TIF) [file pone.0035933.s003.tif]

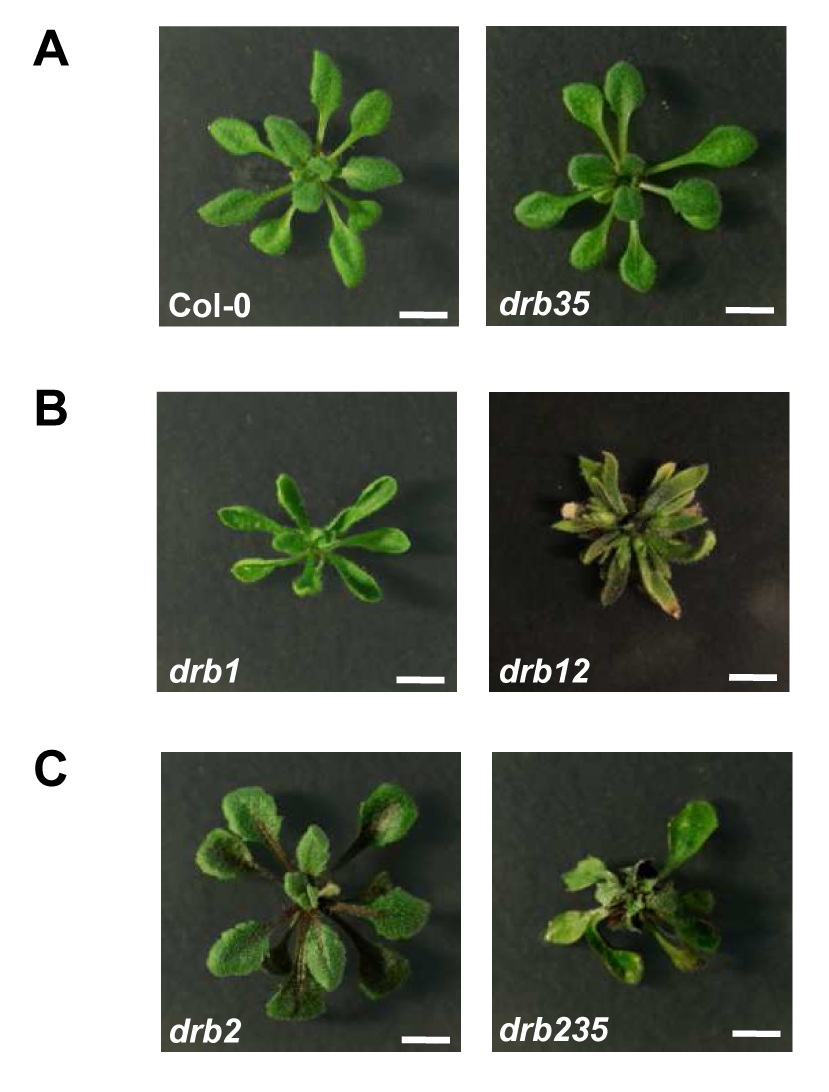

Supplement: Figure S4 — Leaf margin phenotypes displayed by drb mutants with altered miR164, CUC1 and CUC2 levels in the SAM region. (A) Col-0 and drb35 plants display rosette leaves with smooth margins and miR164, CUC1 and CUC2 levels are unchanged. (B) drb1 and drb12 plants have reduced miR164 accumulation and deregulated CUC1 and CUC2 expression and develop rosette leaves with smooth margins. (C) drb2 and drb235 plants develop rosette leaves with highly serrated margins and miR164 accumulation and target gene expression are elevated and reduced respectively in specific tissues of these mutant lines. (A to C) scale bars = 5 mm. (TIF) [file pone.0035933.s004.tif]

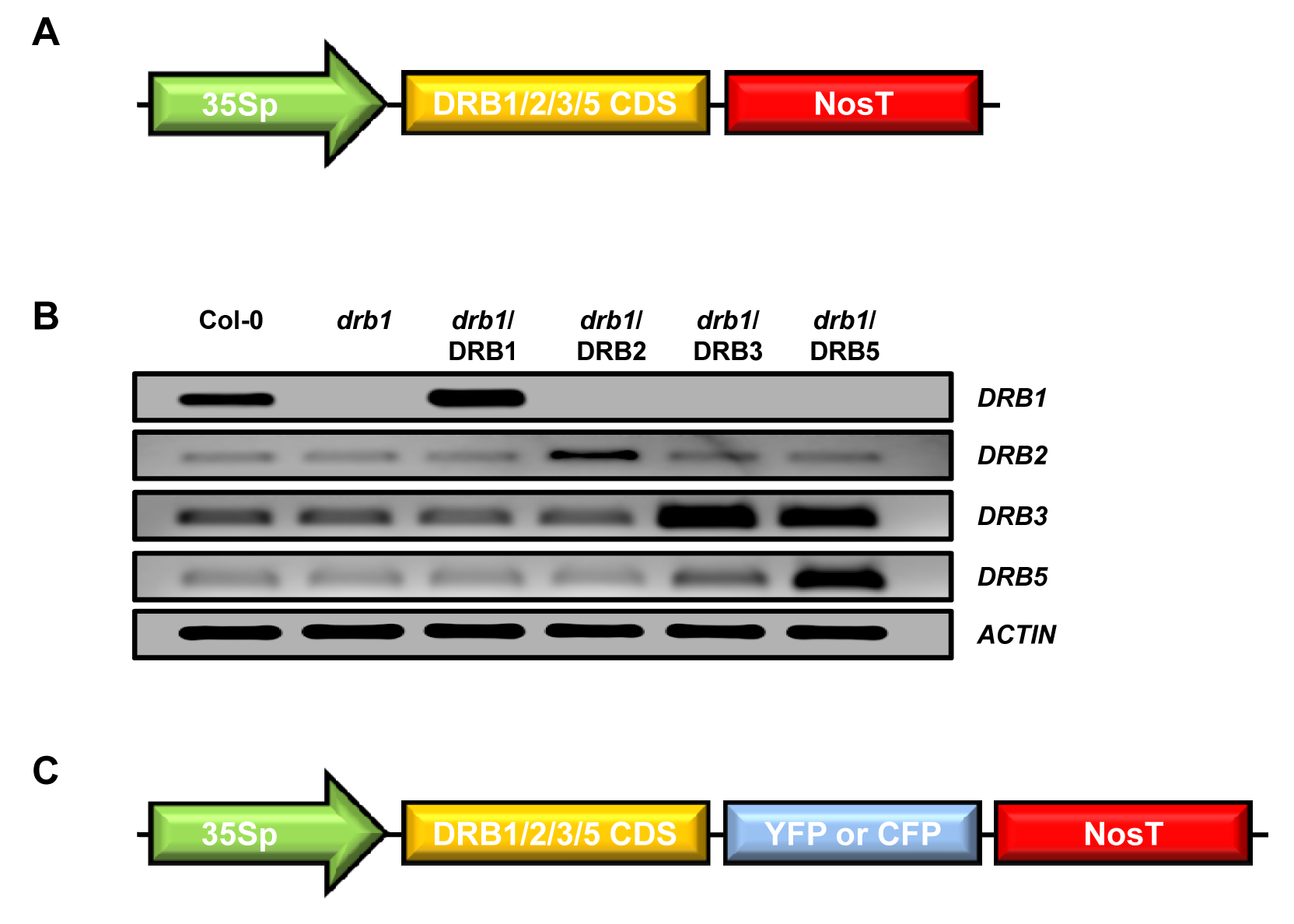

Supplement: Figure S5 — DRB expression in drb1 plants transformed with the DRB1, DRB2, DRB3 and DRB5 over-expression vectors. (A) Schematic of the 35S promoter-driven plant expression vector for the over-expression of DRB1, DRB2, DRB3 and DRB5. (B) RT-PCR analysis of DRB expression in drb1 plants transformed with the DRB1, DRB2, DRB3 and DRB5 over-expression vectors. (C) Schematic of the 35S promoter-driven fluorescent reporter gene vectors developed to visualize the cellular locations of the Arabidopsis DRB1 (CFP), DRB2 (YFP), DRB3 (YFP) and DRB5 (YFP) proteins in Agrobacterium-infiltrated N. benthamiana leaves. (A and C) 35Sp, Cauliflower mosaic virus 35S promoter; DRB1/2/3/5 CDS, DRB coding sequences; NosT, nopaline synthase terminator. (TIF) [file pone.0035933.s005.tif]
