# Supplementary material for: DRB2 Is Required for MicroRNA Biogenesis in Arabidopsis thaliana
Source: PLoS One. 2012 Apr 24;7(4):e35933. doi: 10.1371/journal.pone.0035933 (PMC3335824; doi:10.1371/journal.pone.0035933)
Supplement: Table S1 — miRNA accumulation in the SAM region of drb235 plants. (DOC) [file pone.0035933.s006.doc]

**Table S1.** miRNA accumulation in the SAM region of *drb235* plants.

| **miRNA** | **miRNA** | **Number of Reads** | |  | | **Fold Change** |
| --- | --- | --- | --- | --- | --- | --- |
| **number** | **name** | **Col-0** | ***drb235*** | | **(+/-)** | |
|  |  |  |  | |  | |
| **1** | **miR156a** | 427 | 255 | | 1.7 | |
| **2** | **miR156b** | 428 | 253 | | 1.7 | |
| **3** | **miR156c** | 427 | 255 | | 1.7 | |
| **4** | **miR156d** | 4067 | 2580 | | 1.6 | |
| **5** | **miR156e** | 416 | 245 | | 1.7 | |
| **6** | **miR156f** | 416 | 245 | | 1.7 | |
| **7** | **miR156g** | 18 | 14 | | 1.3 | |
| **8** | **miR156h** | 0 | 0 | | NA | |
| **9** | **miR157a** | 3511 | 3287 | | 1.1 | |
| **10** | **miR157b** | 3511 | 3287 | | 1.1 | |
| **11** | **miR157c** | 3527 | 3302 | | 1.1 | |
| **12** | **miR157d** | 238 | 30 | | -7.9 | |
| **13** | **miR158a** | 2987 | 1860 | | 1.6 | |
| **14** | **miR158b** | 386 | 269 | | 1.4 | |
| **15** | **miR159a** | 19870 | 8566 | | -2.3 | |
| **16** | **miR159b** | 5194 | 2314 | | -2.2 | |
| **17** | **miR159c** | 1746 | 624 | | -2.8 | |
| **18** | **miR160a** | 194 | 64 | | -3.0 | |
| **19** | **miR160b** | 115 | 30 | | -3.8 | |
| **20** | **miR160c** | 114 | 39 | | -2.9 | |
| **21** | **miR161** | 330 | 128 | | -2.6 | |
| **22** | **miR162a** | 2461 | 2528 | | 1.0 | |
| **23** | **miR162b** | 2516 | 2543 | | 1.0 | |
| **24** | **miR163** | 0 | 0 | | NA | |
| **25** | **miR164a** | 148 | 1595 | | +10.7 | |
| **26** | **miR164b** | 148 | 1595 | | +10.7 | |
| **27** | **miR164c** | 139 | 125 | | 1.1 | |
| **28** | **miR165a** | 5685 | 5874 | | 1.0 | |
| **29** | **miR165b** | 5646 | 5844 | | 1.0 | |
| **30** | **miR166a** | 22545 | 20150 | | 1.1 | |
| **31** | **miR166b** | 22477 | 20037 | | 1.1 | |
| **32** | **miR166c** | 22472 | 20036 | | 1.1 | |
| **33** | **miR166d** | 22475 | 20036 | | 1.1 | |
| **34** | **miR166e** | 22338 | 19989 | | 1.1 | |
| **35** | **miR166f** | 22337 | 19983 | | 1.1 | |
| **36** | **miR166g** | 22336 | 19982 | | 1.1 | |
| **37** | **miR167a** | 8610 | 4374 | | -2.0 | |
| **38** | **miR167b** | 8607 | 4374 | | -2.0 | |
| **39** | **miR167c** | 60 | 33 | | 1.8 | |
| **40** | **miR167d** | 693 | 151 | | -4.6 | |
| **41** | **miR168a** | 206 | 195 | | 1.1 | |
| **42** | **miR168b** | 202 | 189 | | 1.1 | |
| **43** | **miR169a** | 3573 | 608 | | -5.9 | |
| **44** | **miR169b** | 71 | 11 | | -6.5 | |
| **45** | **miR169c** | 0 | 0 | | NA | |
| **46** | **miR169d** | 51 | 9 | | -5.7 | |
| **47** | **miR169e** | 43 | 5 | | -8.6 | |
| **48** | **miR169f** | 0 | 0 | | NA | |
| **49** | **miR169g** | 58 | 4 | | -14.5 | |
| **50** | **miR169h** | 0 | 0 | | NA | |
| **51** | **miR169i** | 0 | 0 | | NA | |
| **52** | **miR169j** | 0 | 0 | | NA | |
| **53** | **miR169k** | 0 | 0 | | NA | |
| **54** | **miR169l** | 0 | 0 | | NA | |
| **55** | **miR169m** | 0 | 0 | | NA | |
| **56** | **miR169n** | 0 | 0 | | NA | |
| **57** | **miR170** | 60 | 11 | | -5.5 | |
| **58** | **miR171a** | 1430 | 281 | | -5.1 | |
| **59** | **miR171b** | 32 | 14 | | -2.3 | |
| **60** | **miR171c** | 33 | 14 | | -2.3 | |
| **61** | **miR172a** | 17646 | 5356 | | -3.3 | |
| **62** | **miR172b** | 17646 | 5356 | | -3.3 | |
| **63** | **miR172c** | 778 | 100 | | -7.8 | |
| **64** | **miR172d** | 778 | 100 | | -7.8 | |
| **65** | **miR172e** | 5397 | 3322 | | 1.6 | |
| **66** | **miR173** | 26 | 3 | | -8.7 | |
| **67** | **miR319a** | 2300 | 2173 | | 1.1 | |
| **68** | **miR319b** | 3930 | 3864 | | 1.0 | |
| **69** | **miR319c** | 178 | 111 | | 1.6 | |
| **70** | **miR390a** | 598 | 620 | | 1.0 | |
| **71** | **miR390b** | 598 | 624 | | 1.0 | |
| **72** | **miR391** | 242 | 74 | | -3.3 | |
| **73** | **miR393a** | 17 | 15 | | 1.1 | |
| **74** | **miR393b** | 69 | 64 | | 1.1 | |
| **75** | **miR394a** | 139 | 50 | | -2.8 | |
| **76** | **miR394b** | 139 | 50 | | -2.8 | |
| **77** | **miR395a** | 2321 | 2203 | | 1.1 | |
| **78** | **miR395b** | 1825 | 1309 | | 1.4 | |
| **79** | **miR395c** | 1825 | 1309 | | 1.4 | |
| **80** | **miR395d** | 2322 | 2202 | | 1.1 | |
| **81** | **miR395e** | 2321 | 2202 | | 1.1 | |
| **82** | **miR395f** | 1825 | 1308 | | 1.4 | |
| **83** | **miR396a** | 14483 | 3348 | | -4.3 | |
| **84** | **miR396b** | 13199 | 4164 | | -3.2 | |
| **85** | **miR397a** | 18 | 8 | | -2.3 | |
| **86** | **miR397b** | 0 | 0 | | NA | |
| **87** | **miR398a** | 5001 | 3977 | | 1.3 | |
| **88** | **miR398b** | 15124 | 9513 | | 1.6 | |
| **89** | **miR398c** | 14086 | 13975 | | 1.0 | |
| **90** | **miR399a** | 4730 | 2791 | | 1.7 | |
| **91** | **miR399b** | 4862 | 3016 | | 1.6 | |
| **92** | **miR399c** | 4740 | 2882 | | 1.6 | |
| **93** | **miR399d** | 4122 | 3762 | | 1.1 | |
| **94** | **miR399e** | 431 | 228 | | 1.9 | |
| **95** | **miR399f** | 2320 | 1965 | | 1.2 | |
| **96** | **miR400** | 410 | 175 | | -2.3 | |
| **97** | **miR401** | 0 | 0 | | NA | |
| **98** | **miR402** | 0 | 0 | | NA | |
| **99** | **miR403** | 3902 | 1523 | | -2.6 | |
| **100** | **miR404** | 0 | 0 | | NA | |
| **101** | **miR405a** | 0 | 0 | | NA | |
| **102** | **miR405b** | 0 | 0 | | NA | |
| **103** | **miR405d** | 0 | 0 | | NA | |
| **104** | **miR406** | 0 | 0 | | NA | |
| **105** | **miR407** | 0 | 0 | | NA | |
| **106** | **miR408** | 39568 | 20991 | | 1.9 | |
| **107** | **miR413** | 0 | 0 | | NA | |
| **108** | **miR414** | 0 | 0 | | NA | |
| **109** | **miR415** | 0 | 0 | | NA | |
| **110** | **miR416** | 0 | 0 | | NA | |
| **111** | **miR417** | 0 | 0 | | NA | |
| **112** | **miR418** | 0 | 0 | | NA | |
| **113** | **miR419** | 0 | 0 | | NA | |
| **114** | **miR420** | 0 | 0 | | NA | |
| **115** | **miR426** | 0 | 0 | | NA | |
| **116** | **miR447a** | 11 | 9 | | 1.2 | |
| **117** | **miR447b** | 1 | 1 | | 1.0 | |
| **118** | **miR447c** | 0 | 0 | | NA | |
| **119** | **miR472** | 311 | 208 | | 1.5 | |
| **120** | **miR771** | 0 | 0 | | NA | |
| **121** | **miR773** | 0 | 0 | | NA | |
| **122** | **miR774** | 0 | 0 | | NA | |
| **123** | **miR775** | 135 | 124 | | 1.1 | |
| **124** | **miR776** | 0 | 0 | | NA | |
| **125** | **miR777** | 0 | 0 | | NA | |
| **126** | **miR778** | 0 | 0 | | NA | |
| **127** | **miR779** | 0 | 0 | | NA | |
| **128** | **miR780** | 0 | 0 | | NA | |
| **129** | **miR781** | 0 | 0 | | NA | |
| **130** | **miR782** | 0 | 0 | | NA | |
| **131** | **miR783** | 12 | 32 | | +2.7 | |
| **132** | **miR822** | 929 | 62 | | -15.0 | |
| **133** | **miR823** | 43 | 18 | | -2.4 | |
| **134** | **miR824** | 822 | 880 | | 1.1 | |
| **135** | **miR825** | 0 | 0 | | NA | |
| **136** | **miR826** | 0 | 0 | | NA | |
| **137** | **miR827** | 106 | 96 | | 1.1 | |
| **138** | **miR828** | 0 | 0 | | NA | |
| **139** | **miR829** | 0 | 0 | | NA | |
| **140** | **miR830** | 0 | 0 | | NA | |
| **141** | **miR831** | 0 | 0 | | NA | |
| **142** | **miR832** | 0 | 0 | | NA | |
| **143** | **miR833** | 31 | 45 | | 1.5 | |
| **144** | **miR834** | 0 | 0 | | NA | |
| **145** | **miR835** | 10 | 12 | | 1.2 | |
| **146** | **miR836** | 0 | 0 | | NA | |
| **147** | **miR837** | 24 | 245 | | +10.2 | |
| **148** | **miR838** | 67 | 21 | | -3.2 | |
| **149** | **miR839** | 58 | 6 | | -9.7 | |
| **150** | **miR840** | 0 | 0 | | NA | |
| **151** | **miR841** | 11 | 96 | | +8.7 | |
| **152** | **miR842** | 154 | 41 | | -3.8 | |
| **153** | **miR843** | 0 | 0 | | NA | |
| **154** | **miR844** | 0 | 0 | | NA | |
| **155** | **miR845a** | 0 | 0 | | NA | |
| **156** | **miR845b** | 0 | 0 | | NA | |
| **157** | **miR846** | 46 | 17 | | -2.7 | |
| **158** | **miR847** | 0 | 0 | | NA | |
| **159** | **miR848** | 0 | 0 | | NA | |
| **160** | **miR849** | 0 | 0 | | NA | |
| **161** | **miR850** | 23 | 469 | | +20.4 | |
| **162** | **miR851** | 0 | 0 | | NA | |
| **163** | **miR852** | 33 | 116 | | +3.5 | |
| **164** | **miR853** | 0 | 0 | | NA | |
| **165** | **miR854a** | 0 | 0 | | NA | |
| **166** | **miR854b** | 0 | 0 | | NA | |
| **167** | **miR854c** | 0 | 0 | | NA | |
| **168** | **miR854d** | 0 | 0 | | NA | |
| **169** | **miR855** | 0 | 0 | | NA | |
| **170** | **miR856** | 0 | 0 | | NA | |
| **171** | **miR857** | 178 | 46 | | -3.9 | |
| **172** | **miR858** | 2103 | 664 | | -3.2 | |
| **173** | **miR859** | 0 | 0 | | NA | |
| **174** | **miR860** | 8 | 54 | | +6.8 | |
| **175** | **miR861** | 0 | 0 | | NA | |
| **176** | **miR862** | 0 | 0 | | NA | |
| **177** | **miR863** | 19 | 469 | | +24.7 | |
| **178** | **miR864** | 0 | 0 | | NA | |
| **179** | **miR865** | 10 | 9 | | 1.1 | |
| **180** | **miR866** | 0 | 0 | | NA | |
| **181** | **miR867** | 0 | 0 | | NA | |
| **182** | **miR868** | 0 | 0 | | NA | |
| **183** | **miR869** | 28 | 28 | | 1.0 | |
| **184** | **miR870** | 8 | 10 | | 1.3 | |
| **185** | **miR1886** | 33 | 31 | | 1.1 | |
| **186** | **miR1887** | 0 | 0 | | NA | |
| **187** | **miR1888** | 0 | 0 | | NA | |
| **188** | **miR2111a** | 245 | 316 | | 1.3 | |
| **189** | **miR2111b** | 146 | 128 | | 1.1 | |
|  |  |  |  | |  | |

* miRNA accumulation in *drb235* plants classed as either elevated or reduced if fold change was equal to, or greater than ± 2.0.
