# Supplementary material for: DRB2 Is Required for MicroRNA Biogenesis in Arabidopsis thaliana
Source: PLoS One. 2012 Apr 24;7(4):e35933. doi: 10.1371/journal.pone.0035933 (PMC3335824; doi:10.1371/journal.pone.0035933)
Supplement: Table S3 — The drb235 elevated, unchanged and reduced miRNA accumulation classes. (DOC) [file pone.0035933.s008.doc]

**Table S3.** The *drb235* elevated, unchanged and reduced miRNA accumulation classes.

| | ***MIR* gene** | **Number of reads** | |  | | **Fold change** | | --- | --- | --- | --- | --- | --- | | **family** | **Col-0** | ***drb235*** | | **(+/-)** | | |  |  |  | |  | | | ***Elevated miRNA accumulation class*** | | | | | | |  |  |  | |  | | | ***MIR164*** | 435 | 3315 | | +7.6 | | | ***MIR783*** | 12 | 32 | | +2.7 | | | ***MIR837*** | 24 | 245 | | +10.2 | | | ***MIR841*** | 11 | 96 | | +8.7 | | | ***MIR850*** | 23 | 469 | | +20.4 | | | ***MIR852*** | 33 | 116 | | +3.5 | | | ***MIR860*** | 8 | 54 | | +6.8 | | | ***MIR863*** | 19 | 469 | | +24.7 | | |  |  |  | |  | | | ***Unchanged miRNA accumulation class*** | | | | | | |  |  |  | |  | | | ***MIR156*** | 6199 | 3847 | | 1.6 | | | ***MIR157*** | 10787 | 9906 | | 1.1 | | | ***MIR158*** | 3373 | 2129 | | 1.6 | | | ***MIR162*** | 4977 | 5071 | | 1.0 | | | ***MIR165*** | 11331 | 11718 | | 1.0 | | | ***MIR166*** | 156980 | 140213 | | 1.1 | | | ***MIR168*** | 408 | 384 | | 1.1 | | | ***MIR319*** | 6408 | 6148 | | 1.0 | | | ***MIR390*** | 1196 | 1244 | | 1.0 | | | ***MIR393*** | 86 | 79 | | 1.1 | | | ***MIR395*** | 12439 | 10533 | | 1.2 | | | ***MIR398*** | 34211 | 27465 | | 1.2 | | | ***MIR299*** | 21205 | 14644 | | 1.4 | | | ***MIR408*** | 39568 | 20991 | | 1.9 | | | ***MIR447*** | 12 | 10 | | 1.2 | | | ***MIR472*** | 311 | 208 | | 1.5 | | | ***MIR775*** | 135 | 124 | | 1.1 | | | ***MIR824*** | 822 | 880 | | 1.1 | | | ***MIR827*** | 106 | 96 | | 1.1 | | | ***MIR833*** | 31 | 45 | | 1.5 | | | ***MIR835*** | 10 | 12 | | 1.2 | | | ***MIR865*** | 10 | 9 | | 1.1 | | | ***MIR869*** | 28 | 28 | | 1.0 | | | ***MIR870*** | 8 | 10 | | 1.3 | | | ***MIR1886*** | 33 | 31 | | 1.1 | | | ***MIR2111*** | 391 | 444 | | 1.1 | | |  |  |  | |  | | | ***Reduced miRNA accumulation class*** | | | | | | |  |  |  | |  | | | ***MIR159*** | 26810 | 11504 | | -2.3 | | | ***MIR160*** | 423 | 133 | | -3.2 | | | ***MIR161*** | 330 | 128 | | -2.6 | | | ***MIR167*** | 17970 | 8932 | | -2.0 | | | ***MIR169*** | 3796 | 637 | | -6.0 | | | ***MIR170*** | 60 | 11 | | -5.5 | | | ***MIR171*** | 1495 | 309 | | -4.8 | | | ***MIR172*** | 42245 | 14234 | | -3.0 | | | ***MIR173*** | 26 | 3 | | -8.7 | | | ***MIR391*** | 242 | 74 | | -3.3 | | | ***MIR394*** | 278 | 100 | | -2.8 | | | ***MIR396*** | 27682 | 7512 | | -3.7 | | | ***MIR397*** | 18 | 8 | | -2.3 | | | ***MIR400*** | 410 | 175 | | -2.3 | | | ***MIR403*** | 3902 | 1523 | | -2.6 | | | ***MIR822*** | 929 | 62 | | -15.0 | | | ***MIR823*** | 43 | 18 | | -2.4 | | | ***MIR838*** | 67 | 21 | | -3.2 | | | ***MIR839*** | 58 | 6 | | -9.7 | | | ***MIR842*** | 154 | 41 | | -3.8 | | | ***MIR846*** | 46 | 17 | | -2.7 | | | ***MIR857*** | 178 | 46 | | -3.9 | | | ***MIR858*** | 2103 | 664 | | -3.2 | | |  |  |  | |  | | |  |  |
| --- | --- | --- | --- | --- | --- | --- | --- | --- | --- | --- | --- | --- | --- | --- | --- | --- | --- | --- | --- | --- | --- | --- | --- | --- | --- | --- | --- | --- | --- | --- | --- | --- | --- | --- | --- | --- | --- | --- | --- | --- | --- | --- | --- | --- | --- | --- | --- | --- | --- | --- | --- | --- | --- | --- | --- | --- | --- | --- | --- | --- | --- | --- | --- | --- | --- | --- | --- | --- | --- | --- | --- | --- | --- | --- | --- | --- | --- | --- | --- | --- | --- | --- | --- | --- | --- | --- | --- | --- | --- | --- | --- | --- | --- | --- | --- | --- | --- | --- | --- | --- | --- | --- | --- | --- | --- | --- | --- | --- | --- | --- | --- | --- | --- | --- | --- | --- | --- | --- | --- | --- | --- | --- | --- | --- | --- | --- | --- | --- | --- | --- | --- | --- | --- | --- | --- | --- | --- | --- | --- | --- | --- | --- | --- | --- | --- | --- | --- | --- | --- | --- | --- | --- | --- | --- | --- | --- | --- | --- | --- | --- | --- | --- | --- | --- | --- | --- | --- | --- | --- | --- | --- | --- | --- | --- | --- | --- | --- | --- | --- | --- | --- | --- | --- | --- | --- | --- | --- | --- | --- | --- | --- | --- | --- | --- | --- | --- | --- | --- | --- | --- | --- | --- | --- | --- | --- | --- | --- | --- | --- | --- | --- | --- | --- | --- | --- | --- | --- | --- | --- | --- | --- | --- | --- | --- | --- | --- | --- | --- | --- | --- | --- | --- | --- | --- | --- | --- | --- | --- | --- | --- | --- | --- | --- | --- | --- | --- | --- | --- | --- | --- | --- | --- | --- | --- | --- | --- | --- | --- | --- | --- | --- | --- | --- | --- | --- | --- | --- | --- | --- | --- | --- | --- | --- | --- | --- | --- | --- | --- | --- | --- | --- | --- | --- | --- | --- | --- | --- | --- | --- | --- | --- | --- | --- | --- | --- | --- | --- | --- | --- | --- | --- | --- | --- | --- | --- | --- | --- | --- | --- | --- | --- | --- | --- | --- | --- | --- | --- | --- | --- | --- | --- | --- | --- | --- | --- | --- | --- | --- | --- | --- | --- | --- | --- | --- | --- | --- | --- | --- | --- | --- | --- | --- | --- | --- | --- | --- | --- | --- | --- | --- | --- | --- | --- | --- | --- | --- | --- | --- | --- | --- | --- | --- | --- | --- | --- | --- | --- | --- | --- | --- | --- | --- | --- | --- | --- | --- | --- | --- | --- | --- | --- | --- | --- | --- | --- | --- | --- | --- | --- | --- | --- | --- | --- | --- | --- | --- | --- | --- | --- | --- | --- | --- | --- | --- | --- | --- | --- | --- | --- | --- | --- | --- | --- | --- | --- | --- |

* *MIR* gene family accumulation in *drb235* plants classed as either elevated or reduced if fold change was equal to, or greater than ± 2.0.
