# Supplementary material for: DRB2 Is Required for MicroRNA Biogenesis in Arabidopsis thaliana
Source: PLoS One. 2012 Apr 24;7(4):e35933. doi: 10.1371/journal.pone.0035933 (PMC3335824; doi:10.1371/journal.pone.0035933)
Supplement: Table S4 — DNA oligonucleotide probes used in this study. (DOC) [file pone.0035933.s009.doc]

**Table S4.** DNA oligonucleotide probes used in this study.

| **Probe** |  | **Sequence (5' to 3')** | |
| --- | --- | --- | --- |
|  |  |  | |
| **amiR-PDS** |  | GTGCTTGAATTAAGACCTTAT |  |
| **miR159** |  | TAGAGCTCCCTTCAATCCAAA |  |
| **miR162** |  | CTGGATGCAGAGGTTTATCGA |  |
| **miR164** |  | TGCACGTGCCCTGCTTCTCCA |  |
| **miR165** |  | GGGGCATGAAGCCTGGTCCGA |  |
| **miR168** |  | TTCCCGACCTGCACCAAGCGA |  |
| **miR169** |  | TCGGCAAGTCATCCTTGGCTG |  |
| **miR170** |  | GATATTGACACGGCTCAATCA |  |
| **miR173** |  | GTGATTTCTCTCTGCAAGCGAA |  |
| **miR319** |  | TGGGAGCTCCCTTCAGTCCAA |  |
| **miR390** |  | GGCGCTATCCCTCCTGAGCTT |  |
| **miR822** |  | CATGTGCAAATGCTTCCCGCA |  |
| **miR837** |  | TGAAACGAACAAGAAACTGAT |  |
| **miR839** |  | GGGAACGATGAAAGGTTGGTA |  |
| **miR841** |  | TTCAGTTTCAAGTGGCTCGTA |  |
| **miR850** |  | CTTTGTTGTAGTCCGGATCTTA |  |
| **miR863** |  | ATTGAGATCAACAAGACATAA |  |
| **U6** |  | AGGGGCCATGCTAATCTTCTC |  |
|  |  |  | |
