# Supplementary material for: DRB2 Is Required for MicroRNA Biogenesis in Arabidopsis thaliana
Source: PLoS One. 2012 Apr 24;7(4):e35933. doi: 10.1371/journal.pone.0035933 (PMC3335824; doi:10.1371/journal.pone.0035933)
Supplement: Table S5 — Primers used in this study. (DOC) [file pone.0035933.s010.doc]

**Table S5.** Primers used in this study.

| **Primer name** | **Sequence amplified** | **Sequence (5' to 3')** |
| --- | --- | --- |
|  |  |  |
| **pACTIN-RTF** | *ACTIN* (*AT3G18780*) coding sequence | TCTTCCGCTCTTTCTTTCCA |
| **pACTIN-RTR** |  | GAGAGAACAGCTTGGATGGC |
| **pAGO1-RTF** | *AGO1* (*AT1G48410*) coding sequence | AAGGAGGTCGAGGAGGGTATGG |
| **pAGO1-RTR** |  | GCTGAGAAGACACCGCTTGATAAG |
| **pAMIR-PDS-R** | Modified *PRI-MIR164B* and *PRI-MIR169A* | GTGCTTGAATTAAGACCTTAT |
| **pCFP-XHOI-F** | *CFP* reporter gene coding sequence | TCACTCGAGATGGTGAGCAAGGGCGAGGAG |
| **pCFP-XHOI-R** |  | TCACTCGAGTTACTTGTACAGCTCGTCCATGCCG |
| **pCUC1-RTF** | *CUC1* (*AT3G15170*) coding sequence | GAGGCGTAGTTAGTAGAGAGACGAAC |
| **pCUC1-RTR** |  | AGGAAGAACCGTGGGAGGCAGAGAAGG |
| **pCUC2-RTF** | *CUC2* (*AT5G53950*) coding sequence | GAGTAATTGGGTTATGCATGAATATCG |
| **pCUC2-RTR** |  | GTAGTTCCAAATACAGTCAAGTCC |
| **pCUC3-RTF** | *CUC3* (*AT1G76420*) coding sequence | GAGAGACGACAGGGTTGATT |
| **pCUC3-RTR** |  | TGGCCTCAAGACTAAGTGG |
| **pDRB1-RTF** | *DRB1* (*AT1G09700*) coding sequence | ATGACCTCCACTGATGTTTCCTCT |
| **pDRB1-RTR** |  | TTATGCGTGGCTTGCTTCTGTCTC |
| **pDRB1-OEF** | Construction of the DRB1 over-expression vector | TCAGGTACCATGACCTCCACTGATGTTTCCTCT |
| **pDRB1-OER** |  | TCACCCGGGTTATGCGTGGCTTGCTTCTGTCTC |
| **pDRB1-CFP-R** | Construction of the DRB1*-*CFP expression vector | TCACTCGAGTGCGTGGCTTGCTTCT |
| **pDRB2-RTF** | *DRB2* (*AT2G28380*) coding sequence | ATGTATAAGAACCAGCTACAAGAG |
| **pDRB2-RTR** |  | TCAGATCTTTAGGTTCTCCAGTCG |
| **pDRB2-PRO-F** | Construction of *DRB2* promoter driven *GUS* vector | TCAGGATCCTAATATTTTACTATTCACTCT |
| **pDRB2-PRO-R** |  | TCACCATGGTTCGAAAACCAAATTTACCAA |
| **pDRB2-OEF** | Construction of the DRB2 over-expression vector | TCAGGTACCATGTATAAGAACCAGCTACAAGAG |
| **pDRB2-OER** |  | TCACCCGGGTCAGATCTTTAGGTTCTCCAGTCG |
| **pDRB2-YFP-R** | Construction of the DRB2*-*YFP expression vector | TCACTCGAGGATCTTTAGGTTCTCCAG |
| **pDRB3-RTF** | *DRB3* (*AT3G26932*) coding sequence | ATGTATAAGAATCAGTTGCAAGAG |
| **pDRB3-RTR** |  | CTAATTTGGTAATGACTTCTTCTC |
| **pDRB3-OEF** | Construction of the DRB3 over-expression vector | TCAGGTACCATGTATAAGAATCAGTTGCAAGAG |
| **pDRB3-OER** |  | TCACCCGGGCTAATTTGGTAATGACTTCTTCTC |
| **pDRB3-YFP-R** | Construction of the DRB3*-*YFP expression vector | TCACTCGAGATTTGGTAATGACTTCTTCTC |
| **pDRB5-RTF** | *DRB5* (*AT5G41070*) coding sequence | ATGTATAAGAATCAGCTTCAAGAG |
| **pDRB5-RTR** |  | CTAACTATCATGGGTTTGATCCAA |
| **pDRB5-OEF** | Construction of the DRB5 over-expression vector | TCAGGTACCATGTATAAGAATCAGCTTCAAGAG |
| **pDRB5-OER** |  | TCACCCGGGCTAACTATCATGGGTTTGATCCAA |
| **pDRB5-YFP-R** | Construction of the DRB5*-*YFP expression vector | TCACTCGAGACTATCATGGGTTTGATCC |
| **pM13-F** | pGEM-T Easy cloning vector | TCACACAGGAAACAGCTATGAC |
| **pM13-R** |  | CGCCAGGGTTTTCCCAGTCACGAC |
| **pMYB33-RTF** | *MYB33* (*AT5G06100*) coding sequence | AGTTGTTGTATCCTGGGTGTAGCA |
| **pMYB33-RTR** |  | CCGTTGGTGGTGGTGGAGAC |
| **pMIR164B-F1** | Construction of the pamiR^164B^-PDS vector | TCACTCGAGTGTGTGCATATACGCAAACACA |
| **pMIR164B-F2** |  | GCAAGATATCCTAGAATTAAGTTGCACTTACTAGCTCATA |
| **pMIR164B-F3** |  | TAGTTCCCGCAACTTAAAGCTAGCATATCATGACCACTCC |
| **pMIR164B-R1** |  | TCAAAGCTTGCTGATCAAGATGCGTGATCA |
| **pMIR164B-R2** |  | TAGTAAGTGCAACTTAATTCTAGGATATCTTGCTCATCAC |
| **pMIR164B-R3** |  | TCATGATATGCTAGCTTTAAGTTGCGGGAACTAACTCATC |
| **pMIR169A-F1** | Construction of the pamiR^169A^-PDS vector | TCACTCGAGTCGAATATATATAATTCTTTAATA |
| **pMIR169A-F2** |  | AGTGTGTATCCTAGAATTAAGTTGCACTTTAAATGATCTT |
| **pMIR169A-F3** |  | ACATGAGTGCAACTTGGTTCTAGGATTCACGTTACTTTGT |
| **pMIR169A-R1** |  | TCAGAATTCTTGAGAAGCACGTGTGACGTG |
| **pMIR169A-R2** |  | TTTAAAGTGCAACTTAATTCTAGGATACACACTACTTTCG |
| **pMIR169A-R3** |  | AACGTGAATCCTAGAACCAAGTTGCACTCATGTTACTTTT |
| **pNFYA5-RTF** | *NFYA5* (*AT1G54160*) coding sequence | GAAGATTCATCTTGGGGAAACTC |
| **pNFYA5-RTR** |  | GAGCAGGAAACACAGAGTCTTGA |
| **pPRI-164A-RTF** | *MIR164A* (*AT2G47585*) pri-miRNA transcript | TATAAAGATTTAATCAAC |
| **pPRI-164A-RTR** |  | TCATGTGCTTGGAAATG |
| **pPRI-164B-RTF** | *MIR164B* (*AT5G01747*) pri-miRNA transcript | TGAGCAAGATGGAGAAGCAG |
| **pPRI-164B-RTR** |  | CAGAGGGTATCTACTTCCTC |
| **pPRI-168A-RTF** | *MIR168A* (*AT4G19395*) pri-miRNA transcript | GCCTTGCATCAACTGAAT |
| **pPRI-168A-RTR** |  | CAAACAAAAGGAGACTAAAGA |
| **pPRI-169A-RTF** | *MIR169A* (*AT3G13405*) pri-miRNA transcript | TGGGTATAGCTAGTGAAACGCG |
| **pPRI-169A-RTR** |  | CCTTAGCTTGAGTTCTTGCGA |
| **pTCP4-RTF** | *TCP4* (*AT3G15030*) coding sequence | AACGGAGGAGGGTTTCTGTTC |
| **pTCP4-RTR** |  | GGATTGGTGATGATGGTGAGG |
| **pTUBULIN-RTF** | *TUBULIN* (*AT5G23860*) coding sequence | TGTCAGATCCGGTCCGTACGGTCAG |
| **pTUBULIN-RTR** |  | CACTCATGGTTGCAGAGATGAGGTG |
| **pYFP-XHOI-F** | *YFP* reporter gene coding sequence | TCACTCGAGATGGTGAGCAAGGGCGAGGAG |
| **pYFP-XHOI-R** |  | TCACTCGAGTTACTTGTACAGCTCGTCCATGCCG |
|  |  |  |
