# Supplementary material for: DRB2 Is Required for MicroRNA Biogenesis in Arabidopsis thaliana
Source: PLoS One. 2012 Apr 24;7(4):e35933. doi: 10.1371/journal.pone.0035933 (PMC3335824; doi:10.1371/journal.pone.0035933)
Supplement: Table S6 — Phenotype expression in primary transformant lines generated in this study. (DOC) [file pone.0035933.s011.doc]

**Table S6.** Phenotype expression in primary transformant lines generated in this study.

| **Plant Line** | **Expressing phenotype / N^o^ of**  **primary transformants obtained** | **Percentage (%)** |  |
| --- | --- | --- | --- |
|  |  |  |  |
| **Col-0/DRB2pro:GUS** | 14/17 | 83 |  |
| **Col-0/amiR^164B^-PDS** | 8/8 | 100 | |
| ***drb1*/ amiR^164B^-PDS** | 0/17 | 0 |  |
| ***drb2*/ amiR^164B^-PDS** | 14/14 | 100 | |
| ***drb235*/ amiR^164B^-PDS** | 9/9 | 100 |  |
| **Col-0/amiR^169A^-PDS** | 14/14 | 100 | |
| ***drb1*/ amiR^169A^-PDS** | 0/26 | 0 |  |
| ***drb2*/ amiR^169A^-PDS*** | 13/16 | 81 | |
| ***drb235*/ amiR^169A^-PDS**** | 5/23 | 22 |  |
| ***drb1*/DRB1** | 12/12 | 100 | |
| ***drb1*/DRB2** | 17/18 | 94 |  |
| ***drb1*/DRB3** | 0/23 | 0 | |
| ***drb1*/DRB5** | 0/27 | 0 |  |
|  |  |  |  |

* Number of plant lines with the emergence of green tissues from the SAM region

** Number of plant lines expressing patchy sectors of photo-bleaching
