# Supplementary material for: DRB2 Is Required for MicroRNA Biogenesis in Arabidopsis thaliana
Source: PLoS One. 2012 Apr 24;7(4):e35933. doi: 10.1371/journal.pone.0035933 (PMC3335824; doi:10.1371/journal.pone.0035933)
Supplement: Table S7 — qRT-PCR assessment of precursor transcript and miRNA target gene expression. (DOC) [file pone.0035933.s012.doc]

**Table S7.** qRT-PCR assessment of miRNA precursor transcript and target gene expression.

| **Figure 2A.** | ***drb1*** |  | ***drb2*** |  | ***drb3*** |  | ***drb5*** |  | ***drb23*** |  | ***drb25*** |  | ***drb35*** |  | ***drb235*** | |  | |
| --- | --- | --- | --- | --- | --- | --- | --- | --- | --- | --- | --- | --- | --- | --- | --- | --- | --- | --- |
|  | **Av.** | **St.Dev.** | **Av.** | **St.Dev.** | **Av.** | **St.Dev.** | **Av.** | **St.Dev.** | **Av.** | **St.Dev.** | **Av.** | **St.Dev.** | **Av.** | **St.Dev.** | **Av.** | **St.Dev.** | |  |
|  |  |  |  |  |  |  |  |  |  |  |  |  |  |  |  |  | |  |
| ***PRI-MIR164A*** | 2.05 | ±0.08 | 0.22 | ±0.05 | 0.95 | ±0.10 | 0.97 | ±0.12 | 0.29 | ±0.11 | 0.21 | ±0.09 | 1.05 | ±0.10 | 0.11 | ±0.04 | |  |
| ***PRI-MIR164B*** | 3.18 | ±0.17 | 0.06 | ±0.07 | 0.90 | ±0.06 | 0.89 | ±0.08 | 0.03 | ±0.04 | 0.11 | ±0.05 | 0.89 | ±0.13 | 0.04 | ±0.03 | |  |
| ***CUC1*** | 3.36 | ±0.09 | 0.00 | ±0.00 | 1.02 | ±0.14 | 1.03 | ±0.16 | 0.00 | ±0.00 | 0.00 | ±0.00 | 0.96 | ±0.07 | 0.00 | ±0.00 | |  |
| ***CUC2*** | 4.12 | ±0.13 | 0.15 | ±0.04 | 0.92 | ±0.11 | 0.88 | ±0.11 | 0.21 | ±0.08 | 0.25 | ±0.09 | 0.87 | ±0.04 | 0.00 | ±0.00 | |  |
| ***CUC3*** | 1.11 | ±0.05 | 0.59 | ±0.09 | 1.14 | ±0.19 | 1.13 | ±0.15 | 0.65 | ±0.11 | 0.38 | ±0.21 | 0.96 | ±0.11 | 0.46 | ±0.12 | |  |

| **Figure 2B.** | ***drb1*** |  | ***drb2*** |  | ***drb3*** |  | ***drb5*** |  | ***drb23*** |  | ***drb25*** |  | ***drb35*** |  | ***drb235*** | |  | |
| --- | --- | --- | --- | --- | --- | --- | --- | --- | --- | --- | --- | --- | --- | --- | --- | --- | --- | --- |
|  | **Av.** | **St.Dev.** | **Av.** | **St.Dev.** | **Av.** | **St.Dev.** | **Av.** | **St.Dev.** | **Av.** | **St.Dev.** | **Av.** | **St.Dev.** | **Av.** | **St.Dev.** | **Av.** | **St.Dev.** | |  |
|  |  |  |  |  |  |  |  |  |  |  |  |  |  |  |  |  | |  |
| ***PRI-MIR168A*** | 3.11 | ±0.13 | 1.17 | ±0.07 | 1.12 | ±0.13 | 0.95 | ±0.08 | 0.93 | ±0.15 | 1.06 | ±0.13 | 0.92 | ±0.04 | 1.13 | ±0.09 | |  |
| ***AGO1*** | 2.09 | ±0.24 | 0.97 | ±0.16 | 1.08 | ±0.17 | 1.22 | ±0.18 | 1.14 | ±0.08 | 1.21 | ±0.19 | 0.96 | ±0.15 | 1.17 | ±0.24 | |  |

| **Figure 2C.** | ***drb1*** |  | ***drb2*** |  | ***drb3*** |  | ***drb5*** |  | ***drb23*** |  | ***drb25*** |  | ***drb35*** |  | ***drb235*** | |  | |
| --- | --- | --- | --- | --- | --- | --- | --- | --- | --- | --- | --- | --- | --- | --- | --- | --- | --- | --- |
|  | **Av.** | **St.Dev.** | **Av.** | **St.Dev.** | **Av.** | **St.Dev.** | **Av.** | **St.Dev.** | **Av.** | **St.Dev.** | **Av.** | **St.Dev.** | **Av.** | **St.Dev.** | **Av.** | **St.Dev.** | |  |
|  |  |  |  |  |  |  |  |  |  |  |  |  |  |  |  |  | |  |
| ***PRI-MIR169A*** | 4.45 | ±0.32 | 2.11 | ±0.19 | 0.98 | ±0.09 | 0.96 | ±0.17 | 2.24 | ±0.08 | 2.33 | ±0.16 | 0.89 | ±0.09 | 2.89 | ±0.14 | |  |
| ***NFYA5*** | 3.05 | ±0.10 | 2.09 | ±0.08 | 1.08 | ±0.12 | 1.12 | ±0.21 | 2.88 | ±0.15 | 3.05 | ±0.21 | 1.11 | ±0.05 | 2.77 | ±0.22 | |  |

| **Figure 3A.** | **seedling** |  | **SAM region** | | **Rosette leaves** | | **inflorescence** | | **cauline leaves** | | **floral tissue** | |
| --- | --- | --- | --- | --- | --- | --- | --- | --- | --- | --- | --- | --- |
|  | **Av.** | **St.Dev.** | **Av.** | **St.Dev.** | **Av.** | **St.Dev.** | **Av.** | **St.Dev.** | **Av.** | **St.Dev.** | **Av.** | **St.Dev.** |
|  |  |  |  |  |  |  |  |  |  |  |  |  |
| ***CUC1*** | 0.91 | ±0.15 | 0.00 | ±0.00 | 0.00 | ±0.00 | 0.00 | ±0.00 | 1.00 | ±0.05 | 1.09 | ±0.12 |
| ***CUC2*** | 1.11 | ±0.12 | 0.00 | ±0.00 | 0.94 | ±0.08 | 0.00 | ±0.00 | 1.11 | ±0.19 | 0.87 | ±0.06 |

| **Figure 3B.** | ***drb1*** |  | ***drb2*** |  | ***drb12*** |  | ***drb35*** |  | ***drb235*** |  |
| --- | --- | --- | --- | --- | --- | --- | --- | --- | --- | --- |
|  | **Av.** | **St.Dev.** | **Av.** | **St.Dev.** | **Av.** | **St.Dev.** | **Av.** | **St.Dev.** | **Av.** | **St.Dev.** |
|  |  |  |  |  |  |  |  |  |  |  |
| ***CUC1*** | 3.36 | ±0.09 | 0.00 | ±0.00 | 2.88 | ±0.21 | 0.96 | ±0.07 | 0.00 | ±0.00 |
| ***CUC2*** | 4.12 | ±0.13 | 0.15 | ±0.04 | 3.41 | ±0.16 | 0.87 | ±0.04 | 0.00 | ±0.00 |

| **Figure 4A.** | ***drb1*** |  | ***drb2*** |  | ***drb12*** |  |
| --- | --- | --- | --- | --- | --- | --- |
|  | **Av.** | **St.Dev.** | **Av.** | **St.Dev.** | **Av.** | **St.Dev.** |
|  |  |  |  |  |  |  |
| ***PRI-MIR164A*** | 2.05 | ±0.08 | 0.22 | ±0.22 | 3.01 | ±0.13 |
| ***PRI-MIR164B*** | 3.18 | ±0.17 | 0.06 | ±0.06 | 3.79 | ±0.03 |
| ***CUC1*** | 3.36 | ±0.09 | 0.00 | ±0.00 | 2.88 | ±0.21 |
| ***CUC2*** | 4.12 | ±0.13 | 0.15 | ±0.15 | 3.41 | ±0.16 |

| **Figure 4B.** | ***drb1*** |  | ***drb2*** |  | ***drb12*** |  |
| --- | --- | --- | --- | --- | --- | --- |
|  | **Av.** | **St.Dev.** | **Av.** | **St.Dev.** | **Av.** | **St.Dev.** |
|  |  |  |  |  |  |  |
| ***PRI-MIR168A*** | 3.11 | ±0.13 | 1.17 | ±0.07 | 2.97 | ±0.21 |
| ***AGO1*** | 2.09 | ±0.24 | 0.97 | ±0.16 | 2.01 | ±0.14 |

| **Figure 4C.** | ***drb1*** |  | ***drb2*** |  | ***drb12*** |  |
| --- | --- | --- | --- | --- | --- | --- |
|  | **Av.** | **St.Dev.** | **Av.** | **St.Dev.** | **Av.** | **St.Dev.** |
|  |  |  |  |  |  |  |
| ***PRI-MIR169A*** | 4.45 | ±0.32 | 2.11 | ±0.19 | 5.59 | ±0.09 |
| ***NFYA5*** | 3.05 | ±0.10 | 2.09 | ±0.08 | 4.67 | ±0.16 |

| **Figure 5B.** | ***drb1*** | |  | | ***drb2*** | |  | | ***drb235*** | |  | |
| --- | --- | --- | --- | --- | --- | --- | --- | --- | --- | --- | --- | --- |
|  | **Av.** | | **St.Dev.** | | **Av.** | | **St.Dev.** | | **Av.** | | **St.Dev.** | |
|  |  | |  | |  | |  | |  | |  | |
| ***PRI-MIR164B-PDS*** | | 2.34 | | ±0.11 | | 0.09 | | ±0.05 | | 0.04 | | ±0.02 |
| ***PDS*** | 4.67 | | ±0.24 | | 0.00 | | ±0.00 | | 0.00 | | ±0.00 | |

| **Figure 5D.** | ***drb1*** | |  | | ***drb2*** | |  | | ***drb235*** | |  | |
| --- | --- | --- | --- | --- | --- | --- | --- | --- | --- | --- | --- | --- |
|  | **Av.** | | **St.Dev.** | | **Av.** | | **St.Dev.** | | **Av.** | | **St.Dev.** | |
|  |  | |  | |  | |  | |  | |  | |
| ***PRI-MIR169A-PDS*** | | 5.02 | | ±0.09 | | 2.87 | | ±0.19 | | 3.12 | | ±0.23 |
| ***PDS*** | | 4.09 | | ±0.16 | | 2.56 | | ±0.09 | | 2.91 | | ±0.12 |

| **Figure 6C.** | ***drb1*** |  | ***drb1*/DRB1** | | ***drb1*/DRB2** | | ***drb1*/DRB3** | | ***drb1*/DRB5** | |
| --- | --- | --- | --- | --- | --- | --- | --- | --- | --- | --- |
|  | **Av.** | **St.Dev.** | **Av.** | **St.Dev.** | **Av.** | **St.Dev.** | **Av.** | **St.Dev.** | **Av.** | **St.Dev.** |
|  |  |  |  |  |  |  |  |  |  |  |
| ***CUC2*** | 2.64 | ±0.08 | 1.08 | ±0.11 | 1.15 | ±0.17 | 2.34 | ±0.22 | 2.74 | ±0.19 |
| ***AGO1*** | 1.11 | ±0.06 | 0.97 | ±0.04 | 0.94 | ±0.06 | 1.14 | ±0.08 | 1.19 | ±0.07 |
| ***NFYA5*** | 3.01 | ±0.12 | 0.94 | ±0.07 | 1.09 | ±0.03 | 3.22 | ±0.13 | 3.12 | ±0.06 |

| **Figure S1B.** | ***drb1*** |  | ***drb2*** |  | ***drb3*** |  | ***drb5*** |  | ***drb235*** |  |
| --- | --- | --- | --- | --- | --- | --- | --- | --- | --- | --- |
|  | **Av.** | **St.Dev.** | **Av.** | **St.Dev.** | **Av.** | **St.Dev.** | **Av.** | **St.Dev.** | **Av.** | **St.Dev.** |
|  |  |  |  |  |  |  |  |  |  |  |
| ***MYB33*** | 4.04 | ±0.18 | 1.05 | ±0.04 | 1.09 | ±0.03 | 0.98 | ±0.11 | 1.01 | ±0.09 |
| ***CUC2*** | 3.38 | ±0.07 | 0.94 | ±0.12 | 0.92 | ±0.06 | 1.01 | ±0.05 | 0.91 | ±0.04 |
| ***TCP4*** | 5.51 | ±0.04 | 1.08 | ±0.03 | 1.04 | ±0.05 | 1.09 | ±0.02 | 0.97 | ±0.11 |

| **Figure S5B.** | ***drb1*** |  | ***drb1*/DRB1** | | ***drb1*/DRB2** | | ***drb1*/DRB3** | | ***drb1*/DRB5** | |
| --- | --- | --- | --- | --- | --- | --- | --- | --- | --- | --- |
|  | **Av.** | **St.Dev.** | **Av.** | **St.Dev.** | **Av.** | **St.Dev.** | **Av.** | **St.Dev.** | **Av.** | **St.Dev.** |
|  |  |  |  |  |  |  |  |  |  |  |
| ***DRB1*** | 0.00 | ±0.00 | 11.1 | ±1.08 | 0.00 | ±0.00 | 0.00 | ±0.00 | 0.00 | ±0.00 |
| ***DRB2*** | 1.06 | ±0.04 | 0.98 | ±0.11 | 7.56 | ±0.31 | 1.09 | ±0.08 | 0.94 | ±0.14 |
| ***DRB3*** | 1.14 | ±0.08 | 1.08 | ±0.04 | 1.12 | ±0.07 | 9.12 | ±1.21 | 7.04 | ±0.88 |
| ***DRB5*** | 0.92 | ±0.14 | 0.97 | ±0.08 | 0.91 | ±0.05 | 2.21 | ±0.45 | 6.66 | ±0.97 |
